# Mechanism of Inhibition of the Human Sirtuin Enzyme SIRT3 by Nicotinamide: Computational and Experimental Studies

**DOI:** 10.1371/journal.pone.0107729

**Published:** 2014-09-15

**Authors:** Xiangying Guan, Ping Lin, Eric Knoll, Raj Chakrabarti

**Affiliations:** 1 Division of Fundamental Research, PMC Advanced Technology, LLC, Mount Laurel, New Jersey, United States of America; 2 Department of Chemical Engineering, Carnegie Mellon University, Pittsburgh, Pennsylvania, United States of America; Oak Ridge National Laboratory, United States of America

## Abstract

Sirtuins are key regulators of many cellular functions including cell growth, apoptosis, metabolism, and genetic control of age-related diseases. Sirtuins are themselves regulated by their cofactor nicotinamide adenine dinucleotide (NAD^+^) as well as their reaction product nicotinamide (NAM), the physiological concentrations of which vary during the process of aging. Nicotinamide inhibits sirtuins through the so-called base exchange pathway, wherein rebinding of the reaction product to the enzyme accelerates the reverse reaction. We investigated the mechanism of nicotinamide inhibition of human SIRT3, the major mitochondrial sirtuin deacetylase, *in vitro* and *in silico* using experimental kinetic analysis and Molecular Mechanics-Poisson Boltzmann/Generalized Born Surface Area (MM-PB(GB)SA) binding affinity calculations with molecular dynamics sampling. Through experimental kinetic studies, we demonstrate that NAM inhibition of SIRT3 involves apparent competition between the inhibitor and the enzyme cofactor NAD^+^, contrary to the traditional characterization of base exchange as noncompetitive inhibition. We report a model for base exchange inhibition that relates such kinetic properties to physicochemical properties, including the free energies of enzyme-ligand binding, and estimate the latter through the first reported computational binding affinity calculations for SIRT3:NAD^+^, SIRT3:NAM, and analogous complexes for Sir2. The computational results support our kinetic model, establishing foundations for quantitative modeling of NAD^+^/NAM regulation of mammalian sirtuins during aging and the computational design of sirtuin activators that operate through alleviation of base exchange inhibition.

## Introduction

Many severe diseases often occur later in life (e.g., diabetes, neurodegenerative diseases, cancer, cardiovascular disease, pro-inflammatory diseases, and osteoporosis), indicating that aging is an important risk factor for these conditions [1]. Sirtuins, the highly conserved orthologs of the yeast Sir2 enzyme found in a wide range of organisms ranging from bacteria to man, have been implicated in aging and the regulation of metabolism and genome stability [2], [3]. In mammals, seven sirtuin genes, SIRT1 to SIRT7, have been identified [4], [5]. Human sirtuin type 3 (hereafter referred to as SIRT3 unless otherwise specified), one of the seven mammalian sirtuins thus far identified, is a major mitochondrial protein and has deacetylase activity regulating global mitochondrial lysine acetylation [6], [7]. SIRT3 targets many key metabolic enzymes, including Ac-CS2 (acetyl-CoA synthetase 2) [8], [9], OTC (ornithine transcarbamylase) [10], LCAD (long-chain acetyl-CoA dehydrogenase) [11], and ALDH2 (aldehyde dehydrogenase 2, therefore potentiating fat metabolism during fasting) [12]. In addition, SIRT3 destabilizes the hypoxia-inducible factor HIF-1 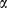, which plays a central role in the Warburg reprogramming of mitochondria – a process that constitutes a critical step in tumorigenesis and aging [13], [14]. Hence, investigation of the mechanism of regulation of SIRT3 activity is of significant importance in connection with mammalian aging.

Most sirtuins are NAD^+^-dependent lysine deacylases, requiring the cofactor NAD^+^ to cleave acyl groups from lysine side chains of their substrate proteins. Mammalian sirtuins have evolved NAD^+^ binding affinities that are low enough that the enzyme activities can be effectively regulated by changes in the physiological concentrations of the cofactor (which according to some reports may range from 200 to 500 µM), allowing them to serve as NAD^+^ sensors [15]. Decreases in NAD^+^ levels that accompany organismic aging can downregulate sirtuin activity [16], [17], whereas increases in NAD^+^ levels that occur due to caloric restriction or NAD^+^ supplementation can upregulate sirtuin activity. Unlike allosteric activators like resveratrol, which are SIRT1-specific and have not been successfully applied to SIRT3 [13], NAD^+^ supplementation can activate most mammalian sirtuins.

Nicotinamide, a well-known water soluble sirtuin inhibitor, is the amide form of vitamin B3 (nicotinic acid), and acts as a constituent of the enzyme cofactors NAD^+^ (nicotinamide adenine dinucleotide) and NADP (nicotinamide adenine dinucleotide phosphate) [18]. NAM is a reaction product and endogenous inhibitor of the deacylation reaction. Physiological NAM levels in some mammalian cells can lie in a range similar to the *IC_50_*'s of several sirtuins [19]–[21], suggesting that some sirtuins may act as NAM sensors as well as NAD^+^ sensors. Mechanistically, NAM binds to a conserved region in the sirtuin catalytic site and favors a reverse base-exchange reaction with an intermediate in the catalytic cycle instead of the deacetylation [22] reaction. An NAM analogue, isonicotinamide (isoNAM), which competes for free NAM binding but does not react appreciably with the intermediate, increases Sir2 activity [23].

Although all sirtuins undergo some level of base exchange inhibition by NAM, it has become increasingly clear that sirtuins have evolved diverse mechanisms for regulation by NAM that are suited to their physiological roles, just as they have evolved different mechanisms for regulation by NAD^+^. For example, Sir2Af2 (Sir2 homolog from *Archaeon Archaeoglobus fulgidus*) is only partially inhibited by NAM, whereas NAM can completely inhibit mSIRT1 [24]. mSIRT3 was reported to display apparent competitive inhibition by NAM, unlike the apparent noncompetitive inhibition reported for other sirtuins [25]. This competitive behavior in NAM inhibition of SIRT3 was conjectured to originate in a mechanism different from the standard base exchange mechanism of SIRT1 [25] and has not yet been explained in terms of the physicochemical properties of the enzyme. Quantitative understanding of the mechanism of inhibition of SIRT3 by NAM is essential for predicting the effects of varying NAD^+^/NAM levels on SIRT3 activity as well as for the rational design of activators that increase SIRT3 activity by alleviation of NAM inhibition. However, due to the sensitivity of NAM regulation kinetics to parameters including the binding affinities of NAD^+^ and NAM, as well as the equilibrium constant for base exchange, the origins of the differences in NAM regulation of sirtuins remain poorly understood.

Available experimental evidence – such as x-ray structures and kinetic assays – is often limited in its ability to explain mechanistic details of sirtuin inhibition by NAM, isoNAM and other inhibitors. Thus far, crystallographic structural analysis of sirtuin binding sites has not been able to explain important differences in binding affinities among sirtuins that play a critical role in their physiological regulation. As will be seen below, evaluation of such binding modes is important for understanding the differences between the mechanisms by which inhibitors exert their effects on SIRT3 and other sirtuins. Computational modeling can evaluate the energetics and intermolecular interactions of binding modes – including unfavorable binding modes – that are difficult to crystallize, through the application of binding affinity estimation methods like the Molecular Mechanics – Poisson Boltzmann (Generalized Born) Surface Area (MM-PB(GB)SA) technique [26]–[29]. Such modeling can elucidate the energetic origins of binding affinity differences, which are often dynamic, more so than a single crystal structure. While such binding affinity estimates do not typically provide accurate absolute *ΔG*'s of binding, they often display significant correlation with experimental binding affinities when used with high-resolution x-ray structures. Finally, the design of novel high affinity and specificity modulators can be aided by the computation of binding affinity estimates for docked ligands.

In this paper, the inhibition mode of NAM for SIRT3 is investigated and compared to that for SIRT1 and Sir2. First, experimental studies of the inhibition kinetics are reported. The mechanism of NAM as a SIRT3 inhibitor is then elucidated through computational studies, including molecular dynamics simulations and binding affinity estimates of NAM and the native NAD^+^ cofactor for SIRT3 and Sir2. A generalized kinetic model for sirtuin base exchange inhibition is introduced and used to explain important differences between the NAM inhibition kinetics of SIRT3 and SIRT1 vis-à-vis the binding of NAD^+^. Unlike prior base exchange inhibition kinetic models for sirtuins, the model we propose relates the physical properties of the enzymes to their degree of competitive inhibition. This is essential for understanding isoform-specific differences in sirtuin inhibition kinetics, which have implications for the physiological and pharmacological regulation of these enzymes. Furthermore, the prospects for upregulating SIRT3 and SIRT1 activity using molecules like isoNAM are investigated. Our computational results support our experimental findings and provide a basis for the computational design of SIRT3-specific modulators.

## Results

### Kinetics of SIRT3/SIRT1 Inhibition by NAM and isoNAM

**Figure 1:**
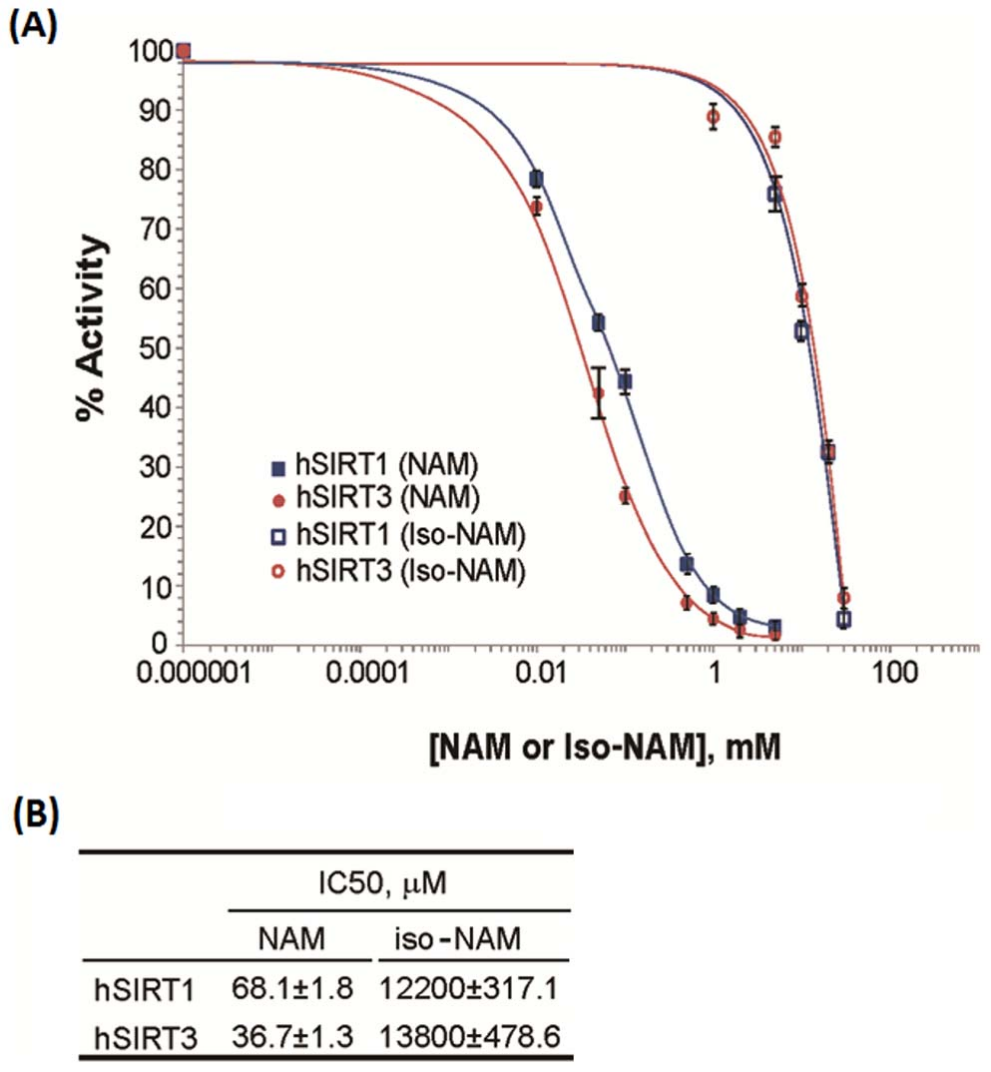
Inhibition of SIRT3/SIRT1 by nicotinamide and its analog. (**A**) Nicotinamide/isonicotinamide inhibition assays showing percent change in deacetylation activity as a function of NAM/isoNAM concentration. Data for the SIRT1 enzyme are indicated with blue squares (filled as NAM, and no filled as isoNAM); data for the SIRT3 enzyme are indicated with red circles (filled as NAM, and no filled as isoNAM). (**B**) The inset table lists the *IC*_50_s of the two inhibitors for these enzymes.

NAM is a known inhibitor of the deacetylation activity of sirtuins, but the inhibition mechanism of NAM has not yet been determined for human SIRT3. In order to compare the inhibitory potency of NAM toward SIRT3 to that of other human sirtuins, we measured its *IC*_50_ value – i.e., the concentration of inhibitor required to cause 50% inhibition under a given assay condition [30]. The inhibition of SIRT3 deacetylation by nicotinamide and isonicotinamide was tested with 90 minutes incubation of 1 mM NAD^+^ at 37°C, providing *IC*_50_ values of 36.7±1.3 µM and 13.8±0.5 mM, respectively. Their *IC*_50_ values for SIRT1 were also measured using the same method. In the case of this enzyme, the *IC*_50_ of NAM is 68.1±1.8 µM and of isoNAM is 12.2±0.3 mM (Fig. 1). These values are in good agreement with reported data [31].

### NAM Acts as a Predominantly Noncompetitive Inhibitor of Recombinant Human SIRT1 and a Mixed Noncompetitive Inhibitor of Recombinant Human SIRT3 *in vitro*

To gain more insight into the effects of NAM on SIRT3 activity, the *in vitro* SIRT3 deacetylation activity was measured in the presence of varying amounts of NAM. We utilized a novel deacetylation activity assay that generates a fluorescent signal upon deacetylation of a peptide substrate. When incubated with acetylated substrate and NAD^+^, recombinant human SIRT3 gives a strong fluorescent signal 10-fold greater than no enzyme and no NAD^+^ controls (data not shown). Using this assay, we tested the ability of nicotinamide to inhibit deacetylation in the presence of varying concentrations of NAD^+^ and saturating concentrations of p53(379–382) peptide substrate. We also measured the *in vitro* SIRT1 deacetylation activity under similar conditions with p53 (317–320) peptide substrate for the purpose of comparison.

The following hyperbolic mixed inhibition initial rate model, derived using steady state assumptions on the various steps of the sirtuin reaction mechanism, can account for the inhibition mechanism of sirtuins wherein NAM engages in a base exchange reaction by reacting with the ADPR-peptidyl intermediate to regenerate NAD^+^ and peptide:
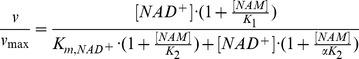
(1)The relationships between *K*_1_, *K*_2_, and *K*_3_≡*αK*_2_ and the rate constants of the sirtuin reaction mechanism are examined in the Discussion.

**Figure 2:**
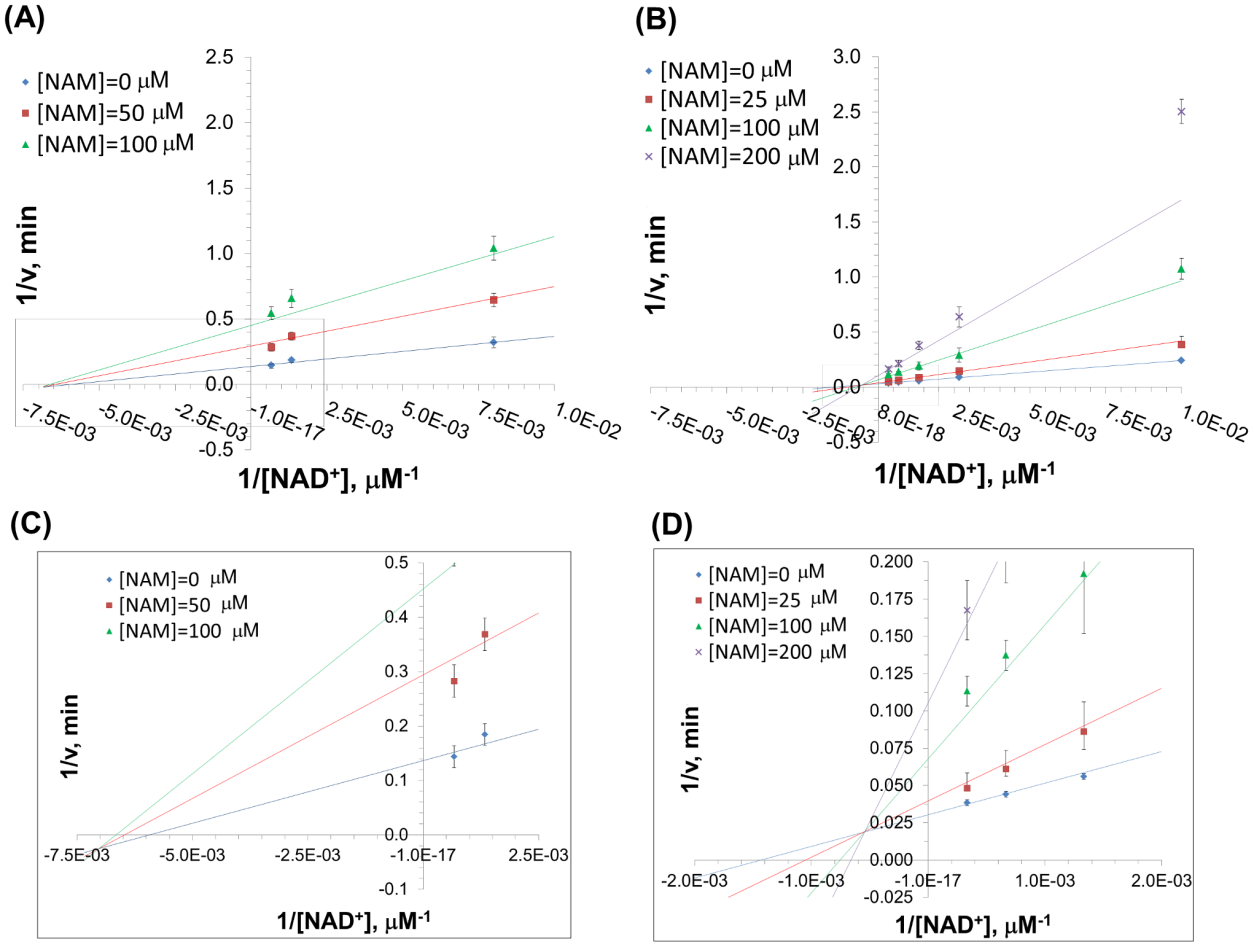
Inhibition of SIRT3/SIRT1 against NAD^+^ by Nicotinamide. (**A**) Recombinant human SIRT1 was incubated for 0, 10, 20, 30, 60, 120, 180, and 240 min at 37°C in the presence of 50, 125, 750, 1500 µM NAD^+^ and 0, 50, and 100 µM NAM. (**B**) Recombinant human SIRT3 was incubated for 0, 10, 20, 30, 60, 120, 180, and 240 min at 37°C in the presence of 100, 375, 750, 1500, 3000 µM NAD^+^ and 0, 25, 100, and 200 µM NAM. Reactions were terminated by the addition of developer and samples were analyzed by fluorometry (excitation set at 355 nm and emission at 460 nm). Data are globally nonlinear fitted to base exchange inhibition model (Eq. 1) and shown as a double-reciprocal plot of 1/*v* versus 1/[*NAD^+^*]. In (**C**) and (**D**), the intersection points of the double reciprocal plots are enlarged for SIRT1 and SIRT3, respectively.

The parameter estimates and associated double reciprocal plots (1/*v vs* 1/[*NAD^+^*]) for global nonlinear fitting of this model to the initial rate data for SIRT3 and SIRT1 inhibition by NAM are provided in Table 1 and Fig. 2, respectively. In both cases, the *K*_1_ was estimated to be sufficiently large that the [*NAM*]/*K*_1_ term in equation (1) could be omitted from the rate expression. Hence *K*_1_ estimates are not included in Table 1.

**Table 1:** Model parameter estimates from global nonlinear fitting of mixed inhibition models for SIRT3 and SIRT1 inhibition by isoNAM (Eq. 2) and NAM (Eq. 1).

	Inhibitor	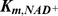 , µM	*V_max_*, µM/min	*K*_2_(*K*_i_)	*α*
SIRT3	isoNAM	1402	0.167	(4623)	8.87E+18
	NAM	673.3	0.197	29.4	2.735
SIRT1	NAM	168.2	0.863	51.3	0.848

**Figure 3:**
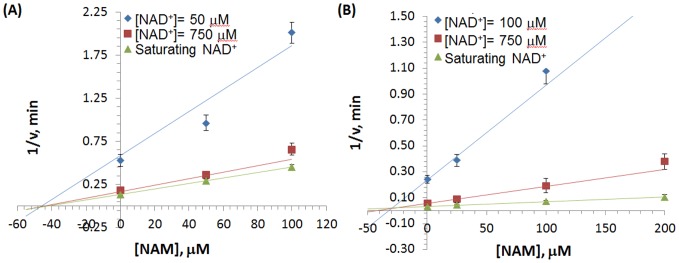
Deacetylation rate as a function of nicotinamide concentration. Dixon plot (1/*v vs* [*NAM*]) of the deacetylation rates for (**A**) SIRT1 and (**B**) SIRT3 enzymes. Experimental data were fit to a linear equation.

For SIRT1, a double reciprocal Lineweaver-Burk plot of the data (Fig. 2A) shows that NAM displays approximately noncompetitive inhibition kinetics (see Discussion for definitions). We next studied the inhibitory mechanism of nicotinamide in the case of SIRT3 *in vitro*. Using SIRT3, we monitored deacetylation of the substrate, as indicated above, in the presence of varying amounts of NAM and NAD^+^. Fig. 2B reveals that NAM inhibition of SIRT3 is mixed noncompetitive with more apparent competition with respect to NAD^+^ than is the case with inhibition of SIRT1. Fig. 3 displays Dixon plots (1/*v vs* [*NAM*]) for various [*NAD^+^*] for both SIRT1 and SIRT3.

### IsoNAM Acts as a Weak Competitive Inhibitor of hSIRT3 with respect to NAD^+^ *in vitro*

Isonicotinamide was reported as an activator of Sir2 activity *in vitro* [23], and this effect was shown to originate through direct competition with nicotinamide for binding. Furthermore, the silencing at telomeres, the HM loci, and the rDNA locus were strengthened by the addition of 25 mM isoNAM to yeast culture, which indicated that Sir2 activity can also be up-regulated *in vivo* [23], [32]–[34]. The effects of isoNAM on SIRT3 activity have not previously been investigated. Here, the *in vitro* SIRT3 deacetylation activity was measured in the presence of varying amounts of isoNAM. Global nonlinear fitting of the following standard mixed noncompetitive inhibition model to initial rate data was carried out for isoNAM inhibition of SIRT3:
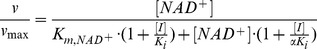
(2)where, [*I*] denotes the concentration of inhibitor (isoNAM). The relationship between *K*_i_ and the rate constants of the sirtuin reaction mechanism is examined in the Discussion.

**Figure 4:**
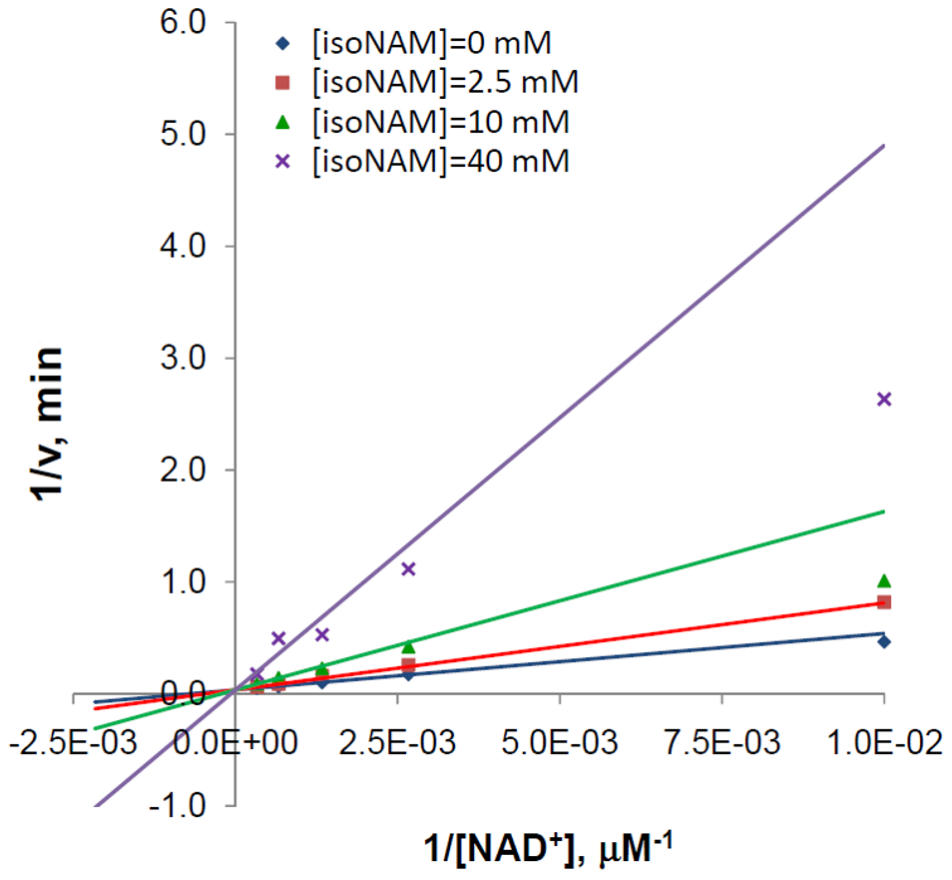
Inhibition of SIRT3 against NAD^+^ by Isonicotinamide. Recombinant human SIRT3 was incubated for 0, 10, 20, 30, 60, 120, 180, and 240 min at 37°C in the presence of 100, 375, 750, 1500, 3000 µM NAD^+^ and 0, 2.5, 10, and 40 mM isoNAM. Reactions were terminated by the addition of developer and samples were analyzed by fluorometry (excitation set at 355 nm and emission at 460 nm). Data are globally nonlinear fitted to mixed inhibition model (Eq. 2) and shown as a double-reciprocal plot of 1/*v* versus 1/[*NAD^+^*].

The double reciprocal representation of the fitting is provided in Fig. 4, and associated parameter estimates are provided in Table 1. The high value of *α* indicates that isoNAM acts as a competitive inhibitor of SIRT3. Since the inhibition was observed to be competitive, and because the *IC_50_* of isoNAM was orders of magnitude higher than that of NAM, the base exchange kinetic model (Eq. 1) was not fit.

### SIRT3 Inhibition by NAM in the Presence of IsoNAM

**Figure 5:**
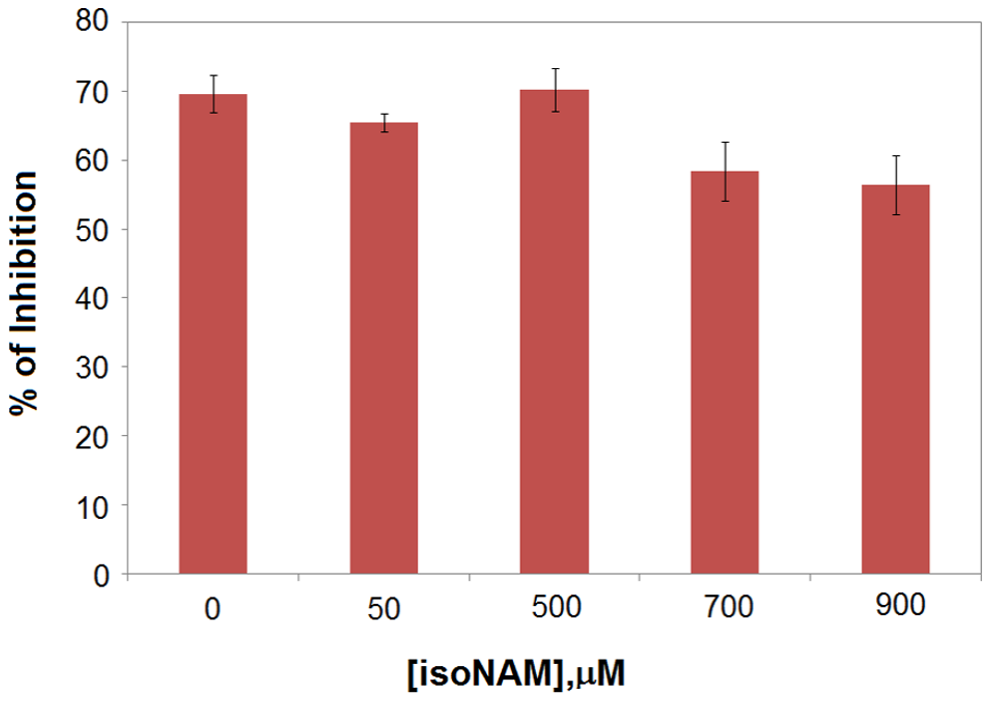
Inhibition of SIRT3 by nicotinamide in the presence of IsoNAM. Recombinant human SIRT3 was incubated with 50, 500, 700 and 900 µM of isoNAM for 40 min at 37°C in the presence of 500 µM NAD^+^, and 100 µM NAM. Reactions were terminated by the addition of developer and samples were analyzed by fluorometry (excitation set at 355 nm and emission at 460 nm).

NAM is a potent inhibitor of the Sir2 deacetylation reaction because of its ability to rebind with the enzyme and react with a high-energy intermediate, preventing deacetylation and regenerating starting materials [24], [35]. As noted above, in the case of some Sir2 enzymes, isoNAM – which does not readily react with the enzyme intermediate – was reported to relieve the inherent NAM inhibition by competitive binding. Prior work on isoNAM inhibition was restricted to Sir2 enzymes. Given the salient differences between mammalian and yeast sirtuins, we investigated whether this derepression effect of isoNAM also applies to SIRT3. The SIRT3 inhibition effect by NAM was studied in the presence of different concentrations of isoNAM. Fig. 5 shows that in the presence of 100 µM NAM, sub-millimolar concentrations (50–900 µM) of isoNAM only slightly decrease the extent of SIRT3 inhibition by NAM – from 69.7% at 0 µM isoNAM to 56.5% at 900 µM isoNAM – under the current experimental conditions (see Methods for details).

### Computational Modeling of NAD^+^ - Sir2 Binding

As a complement to the experimental studies, a computer simulation method involving molecular dynamics (MD) simulations of sirtuin complexes with NAD^+^, NAM, and isoNAM was applied. The MD trajectories were used along with MM-PB(GB)SA scoring function evaluation in order to estimate protein-ligand binding affinities, as described in Methods. Due to the aforementioned differences in NAD^+^ and NAM regulation of mammalian and non-mammalian sirtuins, we carried out computational studies of the above sirtuin-ligand complexes for both bacterial Sir2Tm and hSIRT3. MD simulations capture dynamic contributions to protein-ligand binding that are not apparent from single-structure calculations. Although MM-PB(GB)SA scoring functions, reported in kcal/mol, are not absolute binding affinities, they have been shown to have a significant correlation with experimental binding affinities for many protein-ligand data sets [26]–[29]. The studies by Hou's group [26] suggested that MM-PBSA performed better in predicting absolute binding free energies but that MM-GBSA had better correlation with experiment. Therefore results from both methods are presented.

For both Sir2Tm and SIRT3, high-resolution crystal structures of their ternary complexes with peptide substrates and NAD^+^ or NAD^+^ analogs have been solved. Binding of peptide to sirtuin active sites has been shown to induce domain motions (between Zn-binding domain and the Rossmann fold domain) that are essential to formation of the catalytically active NAD^+^ binding pocket [36], [37], but are difficult to model without the use of long MD simulations. Hence, for computational studies of NAD^+^ binding to Sir2 enzymes, PDB structure 2H4F of the Sir2Tm:Ac-p53:NAD^+^ complex was used, instead of other available structures of Sir2 enzymes such as Sir2Af2 (for which crystal structures of the peptide-bound ternary complexes are not available). Ac-p53 is an 18-residue sequence of 372-KKGQSTSRHK- K[Ac]-LMFKTEG-389 that derives from C-terminal domain of the acetyl-cellular tumor antigen p53 peptide. Due to the lack of availability of a binary SIRT1:peptide complex, analogous computational studies on SIRT1 were not carried out (currently SIRT1 PDB structures are available only in 4I5I [38], 4IG9 and 4KXQ [39]).

Previous work [40] indicated that there are two possible binding sites for NAD^+^ in Sir2 – the so-called AB and AC pockets. AC pocket binding is required for deacetylation [41]. In both binding modes, the ADP-ribose moiety resides in the A pocket. However, the nicotinamide moiety can reside in either the B pocket or C pocket. Early reports on Sir2 inhibition by NAM hypothesized that the apparent noncompetitive inhibition observed in kinetic experiments might be attributable to binding of NAD^+^ in the unproductive AB mode in the presence of NAM, which binds in the C pocket, followed by a conformational rearrangement to the catalytically productive AC binding mode upon dissociation of NAM [42]. Hence we also studied the Sir2Tm:Ac-p53:NAD^+^:NAM complex computationally.

**Figure 6:**
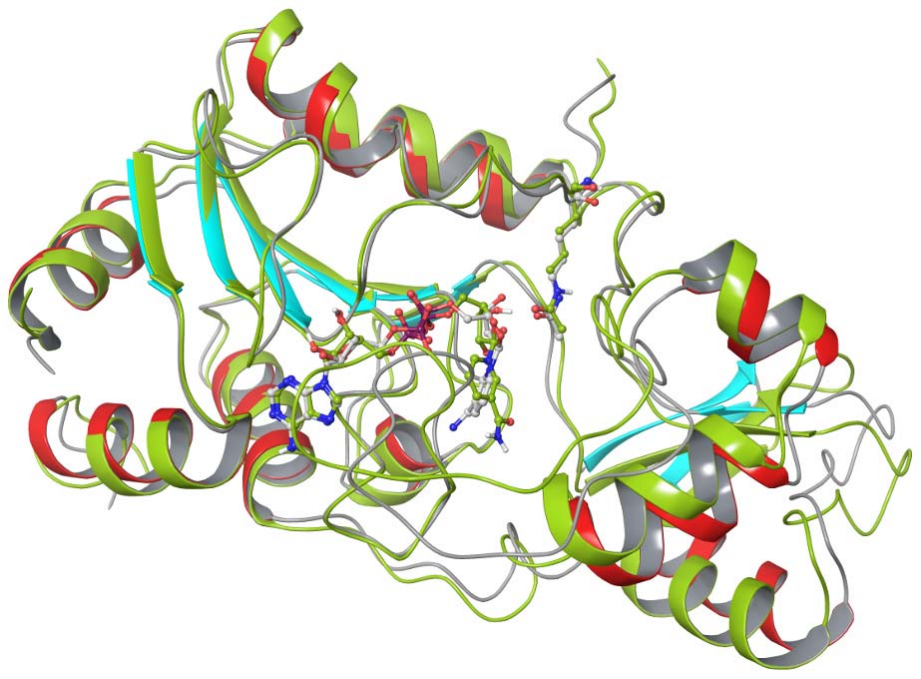
Alignment of ternary complex (Sir2Tm:Ac-p53:NAD^+^) from MD averaged structure with respect to crystal structure. Ribbon representation of crystal structure (PDB ID: 2H4F) is colored by secondary structure (sheets in cyan, helixes in red and coils in gray), MD averaged structure (10 frames over last 10 ps) in yellow green color. Carbon atoms in crystal structure are in white, and are in yellow green for MD averaged structure. Alignment was made using residues 15–27, 182–242, which forms the stable A binding pocket in Rossmann fold domain.

**Figure 7:**
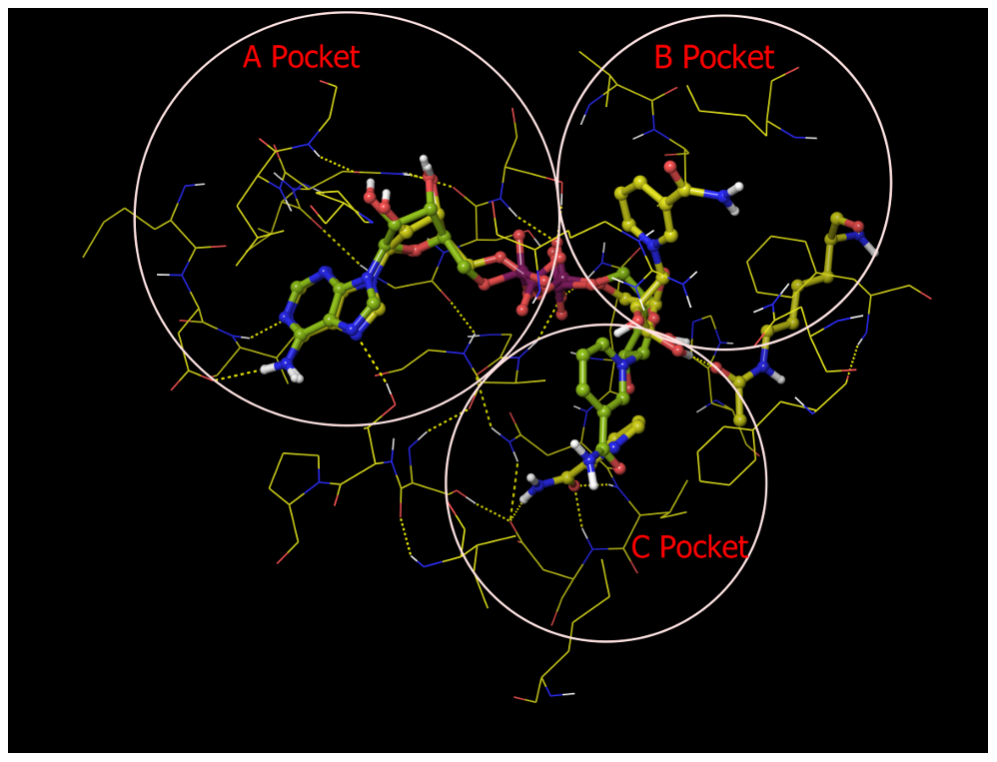
A B, and C pockets of the MD averaged structure of Sir2Tm complex with NAM (Sir2Tm:Ac-p53:NAD^+^:NAM). Carbon is colored in yellow for MD averaged structure of 10 frames over last 10 ps, in yellow green for reference NAD^+^ structure from MD averaged structure over last 10 ps of Sir2Tm ternary complex.

MM-PB(GB)SA binding affinity estimates for NAD^+^ and NAM in these complexes were computed by calculating substrate-receptor interaction energies from a 20 ns MD trajectory for the ternary complex and a 27 ns MD trajectory for the complex with NAM, starting from the complex structures prepared as described in Methods. Fig. 6 compares the structural average of the last 10 ps of the MD trajectory of Sir2Tm:Ac-p53:NAD^+^ to the crystal structure of the ternary complex of Sir2Tm (2H4F) superimposed by alignment of the A pocket. The RMSD of the backbone of these residues between the crystal structure and MD averaged structure is 0.60 Å. The RMSD of NAD^+^ itself (heavy atoms only) after alignment between crystal structure and MD averaged structure is 1.14 Å. Fig. 7 shows a close-up of MD-averaged structure of NAD^+^ adopting the AB binding mode in the presence of NAM.

The MM-PB(GB)SA binding affinity estimates for NAD^+^ in the AC and AB pockets were −23.19±7.27 (−105.48±6.48) kcal/mol and −16.15±8.35 (−81.78±8.01) kcal/mol, respectively (Tables 2 and 3). A breakdown of energetic contributions to NAD^+^ binding is also provided in the tables.

**Table 2:** MD/MM-PB(GB)SA binding affinity estimates for NAD^+^ in SIRT3:Ac-CS2:NAD^+^and Sir2Tm:Ac-p53:NAD^+^ in catalytically productive (without NAM) binding mode.

	SIRT3:Ac-CS2:NAD^+^		Sir2Tm:Ac-p53:NAD^+^	
	Average	Std. Dev.	Average	Std. Dev.
ΔVDWAALS	−68.85	4.82	−78.24	4.71
ΔEEL	−2.14	18.13	−142.87	11.80
ΔEGB	14.13	15.11	125.91	8.05
ΔESURF	−8.76	0.25	−10.28	0.16
ΔEPB	24.47	15.75	152.11	8.21
ΔENPOLAR	−46.14	1.10	−51.62	0.66
ΔEDISPER	87.31	1.58	97.43	0.98
ΔG_gas	−70.99	17.71	−221.11	11.07
ΔG_solv_igb2	5.36	15.11	115.64	8.02
ΔG_solv_pb	65.64	16.00	197.92	8.26
**ΔG_igb2**	**−65.63**	**5.91**	**−105.48**	**6.48**
**ΔG_pb**	**−5.35**	**8.37**	**−23.19**	**7.27**

VDWAALS: van der waals not including the 1–4 terms.

EEL: electrostatic interactions not including the 1–4 terms.

EGB: Polar contribution to solvation energy by GB method.

ESURF: non-polar contribution to solvation energy using SASA (solvent accessible surface area) for GB.

EPB: Polar contribution to solvation energy by PB method.

ENPOLAR: non-polar contribution to solvation energy from repulsive solute-solvent interactions for PB.

EDISPER: non-polar contribution to solvation energy from attractive solute-solvent interactions for PB.

G_gas: Gas phase MM energy including all bonded and non-bonded terms.

G_solv_igb2: Total solvation energy by GB method.

G_solv_pb: Total solvation energy by PB method.

ΔG_igb2: Difference in total energy including gas phase MM energy and solvation energy by GB method.

ΔG_pb: Difference in total energy including gas phase MM energy and solvation energy by PB method.

**Table 3:** MD/MM-PB(GB)SA binding affinity estimates for NAD^+^ in SIRT3:Ac-CS2:NAD^+^ and Sir2Tm:Ac-p53:NAD^+^ in catalytically unproductive (with NAM) binding modes.

	SIRT3:Ac-CS2:NAD^+^:NAM		Sir2Tm:Ac-p53:NAD^+^:NAM	
	Average	Std. Dev.	Average	Std. Dev.
ΔVDWAALS	−72.94	4.35	−72.40	4.57
ΔEEL	52.57	13.53	−82.21	20.85
ΔEGB	−33.94	12.02	81.85	15.75
ΔESURF	−8.81	0.28	−9.03	0.33
ΔEPB	−27.80	12.52	96.05	18.91
ΔENPOLAR	−44.93	1.14	−47.08	1.44
ΔEDISPER	85.00	1.46	89.48	1.83
ΔG_gas	−20.37	13.27	−154.60	21.64
ΔG_solv_igb2	−42.76	12.10	72.82	15.62
ΔG_solv_pb	12.27	12.58	138.45	19.06
**ΔG_igb2**	**−63.12**	**4.37**	**−81.78**	**8.01**
**ΔG_pb**	**−8.10**	**5.71**	**−16.15**	**8.35**

### Computational Modeling of NAD^+^ - SIRT3 Binding

Molecular dynamics simulations and MM-PB(GB)SA binding affinity calculations were also carried out for the SIRT3:NAD^+^, SIRT3:Ac-CS2:NAD^+^, and SIRT3:Ac-CS2:NAD^+^:NAM complexes, where Ac-CS2 denotes a 12-residue sequence of 638-TRSG-K[Ac]-VMRRLLR-649 of acetyl-Co-A synthase 2, in order to investigate the structural and energetic origins of the distinctive features of SIRT3 inhibition kinetics. As described in Methods, the starting structures for these simulations were based on PDB entry 4FVT, which is a ternary complex containing the unreactive NAD^+^ analog carba-NAD^+^ and Ac-CS2.

**Figure 8:**
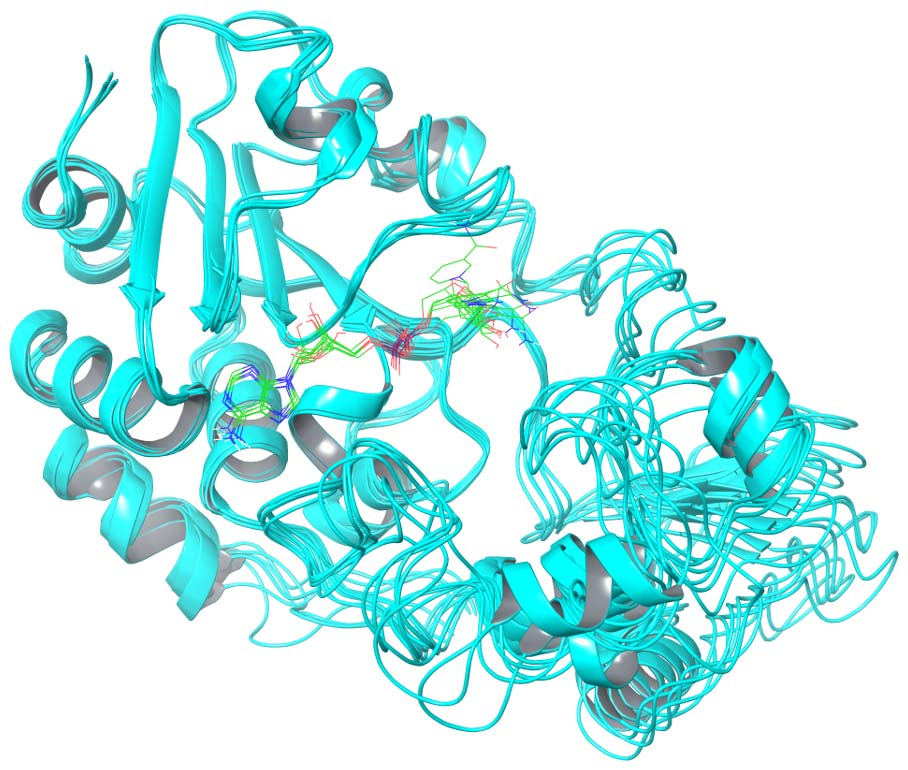
Snapshots taken from MD simulations of SIRT3:NAD^+^ binary complex. Averaged structures of 10 frames over 10 ps at 22, 24, 26, 28, 30 and 32 ns from MD simulation of binary complex (SIRT3:NAD^+^). Alignment was made using residues 139–151, 313–378 in Rossmann fold domain that forms A binding pocket with respect to SIRT3 ternary complex crystal structure 4FVT.

In the case of the binary SIRT3:NAD^+^ complex, the nicotinamide moiety and the Zn binding domain can move significantly over the course of MD when we align the Rossmann fold domain of SIRT3 (Fig. 8). The computed binding affinity did not converge over a 32 ns simulation. This is consistent with reports [37] that peptide binding is required to properly form the NAD^+^ binding pocket in SIRT3.

**Figure 9:**
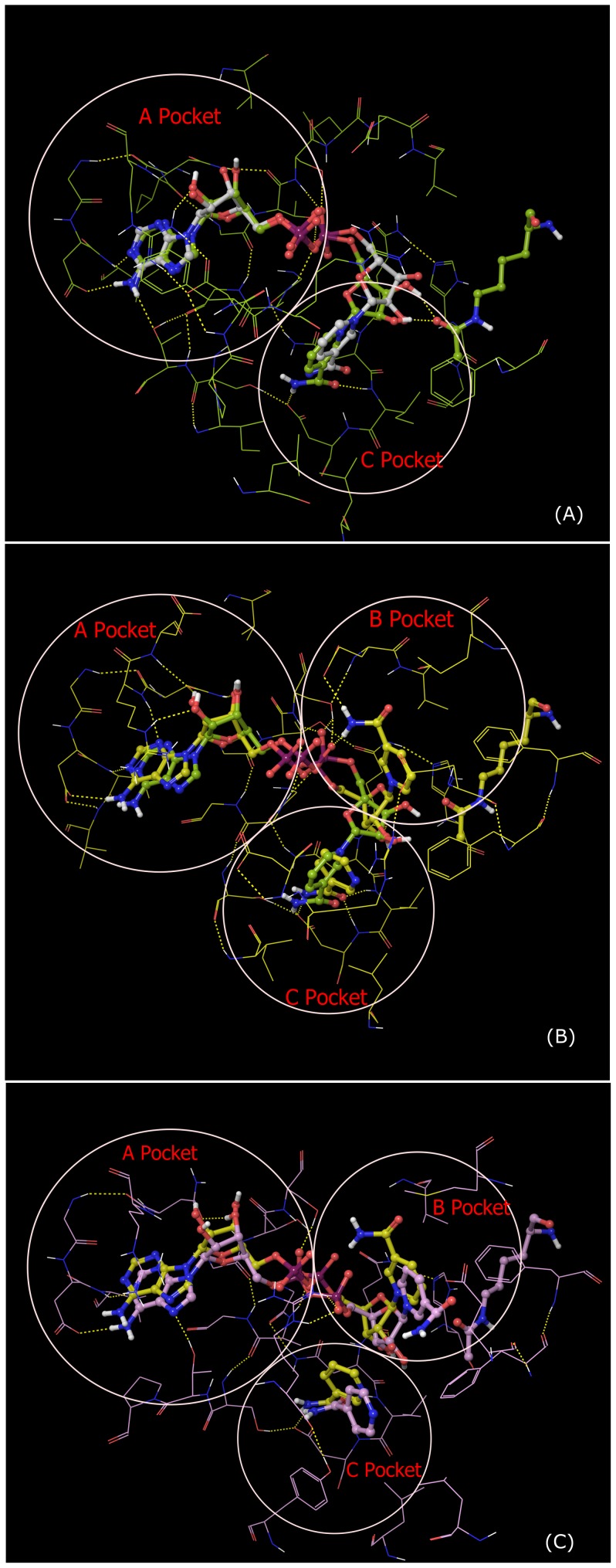
A, B and C binding pockets identified in MD averaged structure of different SIRT3 complexes. (**A**) A and C pockets from the MD averaged structure of 10 frames over last 10 ps of ternary complex (SIRT3:Ac-CS2:NAD^+^, carbon in yellow green), reference NAD^+^ structure (carbon in white) from crystal structure 4FVT; (**B**) A, B and C pockets from the MD averaged structure of 10 frames over last 10 ps of SIRT3 complex with NAM (SIRT3:Ac-ACS2:NAD^+^:NAM, carbon in yellow), reference NAD^+^ structure (carbon in yellow green) from MD averaged structure over last 10 ps of SIRT3 ternary complex; (**C**) A, B and C pockets from the MD averaged structure of 10 frames over last 10 ps of SIRT3 complex with isoNAM (SIRT3:Ac-ACS2:NAD^+^:isoNAM, carbon in plum), reference NAD^+^ structure (carbon in yellow) from MD averaged structure over last 10 ps of SIRT3 complex with NAM.

The SIRT3:Ac-CS2:NAD^+^ starting structure was prepared by converting carba-NAD^+^ into NAD^+^, whereas the SIRT3:Ac-CS2:NAD^+^:NAM starting structure was prepared analogously to the Sir2Tm:Ac-p53:NAD^+^:NAM complex, as described in Methods. Fig. 9 (A) compares the structural average of the last 10 ps of the MD trajectory for SIRT3:Ac-CS2:NAD^+^ (where NAD^+^ is in the catalytically productive binding mode) to the crystallographic coordinates of NAD^+^ (from carba-NAD^+^). The RMSD of NAD^+^ (heavy atoms only) between crystal structure and MD averaged structure is 0.56 Å.

Fig. 9(B) shows a close-up of NAD^+^ adopting the catalytically unproductive binding mode in the presence of NAM. The NAD^+^ in the quaternary complex including NAM is different from the AB pose observed in the crystal structure of Sir2Af2 in complex with NAD^+^ and NAM (as in PDB structure 1YC2 chain A and D) [40]. Although the nicotinamide moiety has similar orientation in the two cases, the ribose linked to the nicotinamide moiety moves closer to the C binding pocket in the SIRT3 quaternary complex with NAM, and the protein-ligand interactions also differ significantly.

The MM-PB(GB)SA binding affinity estimate for NAD^+^ in the SIRT3:Ac-CS2:NAD^+^ complex is −5.35±8.37 (−65.63±5.91) kcal/mol whereas the binding affinity estimate for NAD^+^ in SIRT3:Ac-CS2:NAD^+^:NAM is −8.10±5.71 (−63.12±4.37) kcal/mol (Tables 2, 3). A breakdown of energetic contributions to NAD^+^ binding is also provided in the tables.

### Computational Modeling of NAM/isoNAM Binding to SIRT3 and Sir2

NAM and isoNAM bind in the conserved C pocket of sirtuins. In order to interrogate the possible role that NAM and isoNAM binding affinities may play in their mechanisms of their sirtuin inhibition, we computed MM-PB(GB)SA binding affinity estimates for NAM and isoNAM in SIRT3 and for NAM in Sir2Tm. For NAM, the same MD trajectories reported above for SIRT3:Ac-CS2:NAD^+^:NAM and Sir2Tm:Ac-p53:NAD^+^:NAM were used. The SIRT3:Ac-CS2:NAD^+^:isoNAM complex was prepared analogously to SIRT3:Ac-CS2:NAD^+^:NAM. Fig. 9(C) compares the structural average of the last 10 ps of the MD trajectories for the NAM and isoNAM complexes in SIRT3. The MM-PB(GB)SA binding affinity estimates for NAM in SIRT3 and Sir2Tm were 0.26±3.26 (−19.82±1.89) kcal/mol and −5.78±3.51 (−29.74±2.05) kcal/mol, respectively (Table 4). MM-GBSA calculations (which, as noted, have been reported to display higher correlation with experimental binding affinities) suggest that NAM binds slightly more strongly to the C pocket than isoNAM prior to enzymatic reaction (−19.82 kcal/mol *vs* −15.68 kcal/mol in MM-GBSA scores, respectively). A breakdown of energetic contributions to NAM and isoNAM binding in the C pocket is also provided in the tables.

**Table 4:** MD/MM-PB(GB)SA binding affinity estimates for NAM and isoNAM in SIRT3:Ac-CS2:NAD^+^ and Sir2Tm:Ac-p53:NAD^+^.

	SIRT3:Ac-CS2:NAD^+^:NAM		Sir2Tm:Ac-p53:NAD^+^:NAM		SIRT3:Ac-CS2:NAD^+^:isoNAM	
	Average	Std. Dev.	Average	Std. Dev.	Average	Std. Dev.
ΔVDWAALS	−20.34	1.90	−19.94	1.87	−19.46	2.32
ΔEEL	−24.10	2.84	−18.48	2.95	−11.58	3.69
ΔEGB	27.51	1.64	20.57	1.69	18.10	2.02
ΔESURF	−2.89	0.08	−2.88	0.08	−2.75	0.16
ΔEPB	33.96	2.84	22.49	2.98	23.94	2.93
ΔENPOLAR	−14.47	0.25	−14.44	0.28	−13.49	0.59
ΔEDISPER	25.20	0.59	24.60	0.64	24.47	0.93
ΔG_gas	−44.44	2.59	−38.42	2.84	−31.04	3.67
ΔG_solv_igb2	24.62	1.63	17.69	1.67	15.35	2.01
ΔG_solv_pb	44.70	3.20	32.65	3.15	34.92	3.33
**ΔG_igb2**	**−19.82**	**1.89**	**−20.74**	**2.05**	**−15.68**	**2.48**
**ΔG_pb**	**0.26**	**3.26**	**−5.78**	**3.51**	**3.89**	**4.18**

## Discussion

SIRT3 is a mitochondrial deacetylase protein that can regulate a number of cellular processes, including apoptosis, growth, and metabolism [43]. Moreover, SIRT3 has tumor suppressive functions and reduces the glycolytic metabolism. Cancer initiation and progression depend on aerobic glycolysis, by which cancer cells synthesize biomass for their rapid growth [13]. On the other hand, for normal tissue, downregulation of SIRT3 by NAM would increase glycolytic metabolism and allow cells in impacted tissues to survive longer, reducing long-term tissue damage. Understanding the properties of the inhibitory mechanism of SIRT3 will help elucidate the mechanism of SIRT3-mediated deacetylation and allow improvements in the design of selectivity and affinity of both inhibitors and activators.

In the so-called “NAD^+^ world” picture of global metabolic regulation, the relative concentrations of the sirtuin cofactor NAD^+^ and the sirtuin inhibitor NAM – which can vary with age – play a central role in regulating mammalian metabolism through sirtuin-dependent pathways [44]. For example, SIRT3 regulation by NAD^+^ levels in mitochondria was shown to be a primary determinant of cellular resistance to apoptosis [45]. The average ratio of NAD^+^ to NAM concentrations in a cellular subcompartment can decrease with age, reducing sirtuin activities, due to factors including consumption of NAD^+^ by the NAD^+^-dependent PARP DNA repair enzymes [17] and reduced levels of NAD^+^ synthesis from NAM by the Nampt biosynthetic pathway [44].

NAM is the physiological sirtuin inhibitor. The *IC*_50_ values for nicotinamide inhibition of bacterial Sir2, yeast Sir2, mouse Sir2, SIRT1, SIRT2, SIRT3, and SIRT5 were measured to be 26, 120, 160, 50, 100, 36.7 µM, and 1.6 mM respectively [24], [46]–[48]. Nuclear NAM levels in yeast have been estimated to be 10–150 µM [23], which most likely make NAM a Sir2 activity regulator *in vivo*. Sir2 thus appears to be affected by physiological NAM concentrations, and a role of NAM as an endogenous Sir2 regulator has been supported by *in vivo* studies in yeast and flies [15], [49]. In mammals, low levels of NAM have been measured in several rat tissues, probably as a result of its rapid utilization in the synthesis of NAD^+^ and other pyridine nucleotides [50]. However, NAM concentrations as high as 300 µM have been reported in the brain of Tg2576 mice, providing evidence that NAM concentrations can play a role in regulating sirtuin activities in mammalian cells [51].

Mammalian sirtuins have evolved diverse strategies for regulation by NAD^+^ and NAM based on the relative magnitudes of their binding affinities for these molecules as well as the values of their base exchange equilibrium constants [48]. Due to the interplay of effects of NAD^+^ and NAM binding in sirtuin regulation, mechanistic modeling of inhibition is essential for prediction of the effects of varying NAD^+^/NAM ratios on sirtuin activity. For example, due to their low *K*_m_'s for NAD^+^, yeast and bacterial sirtuins may be less sensitive to regulation by NAD^+^ concentration than mammalian sirtuins [21], [49]. Hence increase in [*NAD^+^*] above average physiological levels in a subcompartment can effectively “activate” mammalian sirtuins [52] but may not have a pronounced effect on yeast/bacterial sirtuins. Moreover, the higher *K_m_*'s and *K_d_*'s of NAD^+^ for mammalian sirtuins are expected to result in important differences in their kinetics of inhibition by NAM. The physiological importance of SIRT3 regulation by NAD^+^ and NAM, and our experimental observation that NAM inhibition of SIRT3 is more competitive toward NAD^+^ than is inhibition of SIRT1, motivate a detailed study of the inhibitory mechanism of SIRT3.

### Kinetic Model for Inhibition of SIRT3 by NAM

**Figure 10:**
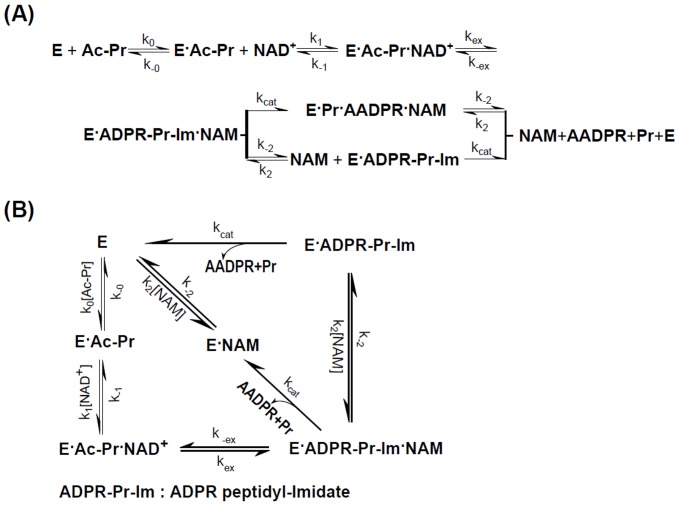
General model for the inhibition of sirtuins by NAM. (**A**) Reaction scheme for the sirtuin deacetylation reaction and base exchange inhibition. For simplicity, deacetylation and AADPR+Pr dissociation are assumed to occur together (*k_cat_* denotes the rate constant for deacetylation and dissociation of AADPR+Pr from E). Binding of NAM to E.Ac-Pr is not depicted in this scheme since base exchange is responsible for NAM inhibition of sirtuins and direct competition between NAM and NAD^+^ can be neglected, as discussed in the text. (**B**) Simplified reaction network for base exchange inhibition. In the presence of saturating Ac-Pr, E is rapidly converted into E^.^Ac-Pr and NAM binding to E can be neglected, resulting in a simplified reaction network with 5 species. ADPR, adenosine diphosphate ribose; AADPR, O-acetyl-adenosine-diphosphate-ribose.

In the sirtuin catalytic cycle (Fig. 10), NAM can react to regenerate acetyl-lysine and NAD^+^ in a nicotinamide exchange reaction with the alkylimidate intermediate [24], [40], [46]. It has been observed that NAM inhibition of Sir2-mediated deacetylation depends on this ability to condense with the high-energy enzyme:ADP-Ribose:acetyl-lysine intermediate to reverse the reaction, reforming NAD^+^ [15]. By using [carbonyl-^14^C] NAM, the base exchange reaction for Sir2 was extensively studied [24]. When NAM is bound to the C pocket, the intermediate can undergo either the base exchange reaction (a backward *β*-face nucleophilic process, resulting in inhibition of deacetylation), or deacetylation (a *α*-face nucleophilic process). Hence the intermediate-forming step is involved in both the exchange and deacetylation reactions, and the branching ratio is determined by the relative rates of the chemical processes.

A common model used to fit enzyme inhibition kinetics is mixed noncompetitive inhibition [53]. For example, equation (2) is a standard mixed noncompetitive inhibition model suitable for describing the kinetics of inhibition by unreactive ligands in the presence of saturating peptide and varying NAD^+^ substrate concentrations. Mechanistically, mixed noncompetitive inhibition with nonzero 1/*α* can originate through a) the inhibitor binding to both the free enzyme and enzyme-substrate complex (in the case that the inhibitor inhibits the forward reaction), b) promotion of the reverse reaction via binding of a reaction product to the enzyme or an enzyme-intermediate complex, or c) a combination of both. If *α*>>1, mixed noncompetitive inhibition becomes competitive inhibition. Here, the apparent *K_m_* of the substrate is changed by the inhibitor, but the *v_max_* is not. If *α* = 1, the inhibition mode is said to be noncompetitive. In this case, the apparent *K_m_* is not altered by the inhibitor, but *v_max_* is. If *α*<<1, the inhibition mode is called uncompetitive, and both *K_m_* and *v_max_* change by the same amount.

Base exchange inhibition of sirtuins, which falls into mixed noncompetitive category b) above, has traditionally been characterized as noncompetitive inhibition without analysis of the extent of effective competition between NAM and the sirtuin cofactor NAD^+^. Our experimental observation (Fig. 2 and Table 1) that human SIRT3 inhibition by NAM displays a greater degree of competitive behavior than SIRT1 demands a mechanistic explanation in terms of the physicochemical properties of the enzymes. Jin et al. made a similar observation for mouse SIRT3 inhibition by NAM reporting *α*>>1, which is indicative of predominantly competitive inhibition [37]. A mechanistic explanation is not possible on the basis of current simplified models of sirtuin base exchange inhibition, which do not relate inhibition constants to enzyme physicochemical properties. Here we introduce such a mechanistic model for base exchange inhibition that is capable of explaining the range of observed mixed noncompetitive inhibition observed in sirtuin base exchange.

An initial rate model for NAM base exchange inhibition was provided in equation (1). Note that this model has features that differ from those of the standard mixed noncompetitive inhibition model (2). Analysis of the sirtuin reaction scheme, shown in Fig. 10, is necessary for derivation of the explicit expressions for the inhibition constants in this equation.

Fig. 10 depicts acetylated peptide binding occurring prior to NAD^+^ binding in an ordered bireactants model. This is supported by the SIRT3:NAD^+^ binary complex molecular dynamics data, which show very weak binding of NAD^+^ to SIRT3 and highly dynamic fluctuations of the nicotinamide moiety of the ligand in the absence of peptide (Fig. 8). Under saturating peptide concentrations, nearly all E is converted rapidly to E^.^Ac-Pr, and hence both E and the step E→E^.^NAM can also be omitted from the reaction scheme. (Previously reported experimental measurements [54], [55] verify that the binding affinity of Ac-Pr is higher than that of NAD^+^ for the types of peptide substrates and sirtuin enzymes considered in this work, and that the peptide concentrations employed in our kinetic studies are saturating.) The catalytic chemistry following the formation of the ADPR-Pr-Im (alkylimidate) intermediate has previously been modeled using mixed quantum/molecular mechanics (QM/MM) simulations in non-mammalian sirtuins [56]. *k_cat_* in our model can be interpreted as the rate constant of the rate limiting step of this catalytic chemistry and the dissociation of AADPR+Pr (which for simplicity is assumed to occur concurrently with deacetylation).

Applying a steady-state analysis to the reaction scheme in Fig. 10 (B) results in the initial rate model (1), where *K_1_*, *K_2_*, and *K_3_*≡*αK_2_* are functions of the rate constants in the Fig.10 (B) that can be obtained by solution of the linear system of algebraic steady state conditions and mass balance constraints. A full analysis including derivation of the exact expressions for these constants will be reported in a separate work. Under the approximation where *k_cat_*<<*k_j_*, *j≠cat*, it can be shown that *K_1_≈K_d, NAM_*, 
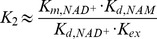
, 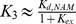, and hence
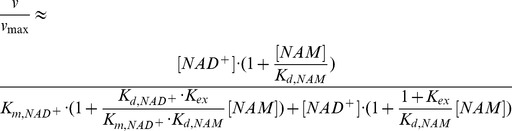
(3)where 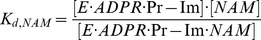. We do not explore the accuracy of the aforementioned approximation in the present work. (For sirtuins, *k_cat_*<<*k_j_*, *j≠cat* does not imply 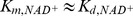.).

Due to the diagonal path in Fig. 10(B) (equivalently, the upper branch in Fig. 10(A)), base exchange inhibition for sirtuins is in fact hyperbolic mixed inhibition [53] – where the inhibitor affects the numerator of the initial rate expression (3) as well as its denominator – since deacetylation can occur from both NAM-bound and dissociated complexes. The fact that the NAM-bound complex is catalytically active results in a plateau in 1/*v vs.* [*NAM*]. The maximum possible extent of base exchange product inhibition at saturating [*NAD^+^*] can be found as follows. At saturating [*NAD^+^*],
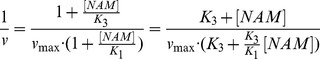
(4)When [*NAM*] is also saturating, the remaining activity, expressed as a fraction of maximal rate (*v_max_*) in absence of inhibitor at saturating [*NAD^+^*], is thus given by *K_3_*/*K_1_*≈1/(1+*K_ex_*) where the approximate equality holds in the case that *k_cat_*<<*k_j_*, *j≠cat*. Since SIRT3 and SIRT1 can both be nearly completely inhibited by NAM, this implies *K_1_*>>*K_3_*, which was confirmed by the results of global nonlinear fitting of equation (1) for these enzymes and the linearity of the Dixon plots in Fig. 3 at the NAM concentrations tested. Thus *K_ex_*>>1 for both SIRT3 and SIRT1.

**Figure 11:**
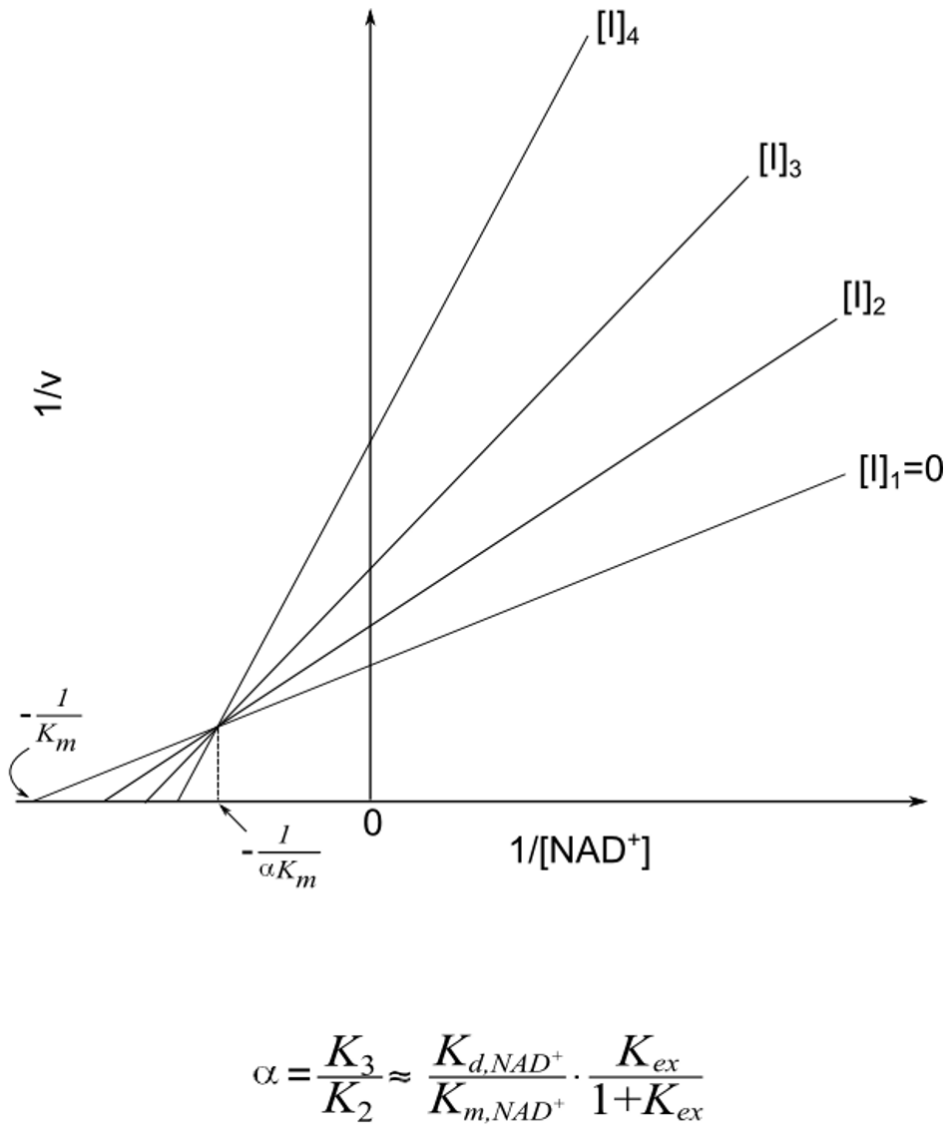
Mixed noncompetitive base exchange inhibition kinetics of sirtuin enzymes: mechanistic interpretation.

Due to the large value of *K_ex_* and hence *K_1_*, it can be shown that at sufficiently low [*NAM*] the plots of 1/*v vs* 1/[*NAD^+^*] at various [*NAM*] intersect at a single point with x-coordinate
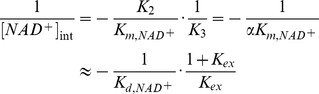
(5)with
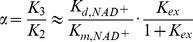
(6)where the approximate equalities again refer to the case *k_cat_*<<*k_j_*, *j≠cat*. Fig. 11 graphically depicts the definition of 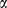 for base exchange inhibition.

Hence a higher ratio of the dissociation constant of NAD^+^ to the Michaelis constant of NAD^+^ is expected to result in a higher value of *α*, as is observed with SIRT3 (Table 1). Based on this analysis, we see how physical properties of a sirtuin affect its value of *α*. The greater competitive behavior in the inhibition kinetics of SIRT3 is also visible in the Dixon plots of Fig. 3; more competitive behavior leads to a smaller slope of the 1/*v_max_ vs* [*NAM*] line at saturating [*NAD^+^*]. Based on equation (4), given that *K_1_*>>*K_3_*, at saturating [*NAD^+^*], the slope of the Dixon plot is approximately equal to 
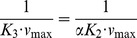
. Hence a higher value of *α*, which means increased competitive behavior in the inhibition kinetics, decreases the slope of the Dixon plot. Note that under the approximation *k_cat_*<<*k_j_*, *j≠cat*, the x-coordinate of the intersection point of the double reciprocal plots at various [*NAM*] provides an estimate of 
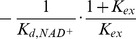
 according to equation (5); hence a higher 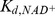 of a sirtuin is expected to result in an intersection point closer to the *y*-axis. (These equations are not intended to provide an accurate estimate of 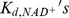, but rather their relative magnitudes, due to the aforementioned approximations.)

According to equation (3), base exchange product inhibition can amplify the effect of the inhibitor binding through *K_ex_* and produce potent inhibition even if the inhibitor binds only weakly to the active site. If the concentrations of substrate and product are of the same order of magnitude (as they often are in physiological regulation of sirtuins), but the *K_d_* of product is of a larger order of magnitude, potent inhibition requires reverse rate constants (for sirtuins, *k_−ex_*) to be suitably large.

NAM inhibition of sirtuin enzymes can thus exhibit a wide range of potent mixed noncompetitive inhibition behavior depending on the values of 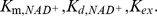 In particular, SIRT3's may exhibit NAM base exchange inhibition kinetics that are apparently more competitive than that of SIRT1, due to a higher ratio 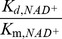.

Jin et al. suggested that NAM inhibition of mSIRT3 may occur through a mechanism different from that of SIRT1 and Sir2, due to its more highly competitive kinetics [37]. We will show below, however, that computationally estimated binding affinities of NAD^+^ and NAM to SIRT3 and Sir2 are consistent with mechanistic model (3) and equation (6), which explain the differences in competitive behavior in sirtuin inhibition by NAM in terms of base exchange and differences in the magnitudes of the physical constants appearing in the sirtuin reaction mechanism depicted in Fig. 10. To date, computational modeling of sirtuin catalytic mechanisms has focused on mixed quantum/molecular mechanics simulations of reactive chemistry in non-mammalian sirtuins [57], [58]. However, sirtuin regulation by NAD^+^/NAM in mammals is heavily dependent on the enzyme's binding affinity for these molecules. To our knowledge, there have been no reports of computational studies of sirtuin inhibition by NAM addressing the critical binding events in the reaction mechanism, and no structural energetic analysis analyzing the causes of the often substantially different values of 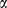 observed among sirtuins. Moreover, there have been few if any direct measurements of 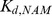 for sirtuins. In order to fill this gap and establish the roles of 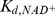 and 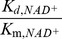 in determining the properties of the double reciprocal plot intersection point (Fig. 11) for sirtuin inhibition by NAM, we undertook a detailed computational analysis of the energetics of the binding affinities of various complexes in the sirtuin reaction mechanism, as well as complexes involving isoNAM, critically evaluating various hypotheses regarding the origin of the greater competitive behavior in the inhibition kinetics of SIRT3 by NAM.

### Binding Affinities of NAM and isoNAM to SIRT3, and the SIRT3 Base Exchange Constant

Equation (3) shows that under the approximation *k_cat_*<<*k_j_*, *j≠cat*, three quantities besides 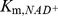 contribute to the observed kinetics of SIRT3 inhibition by NAM:*K_d,NAM_*, *K_ex_*, and 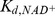. We first sought to estimate the *K_d,NAM_*, through the use of computational binding affinity estimates for NAM and its structural isomer isoNAM together with experimental estimates of the dissociation constant *K_d,isoNAM_*.

Defining inhibition modality is necessary for calculating the enzyme-inhibitor dissociation constant from the experimental assays [59]. For isoNAM inhibition, *α*>>1 and *K_i_*≈4.6 mM – much higher than that of NAM (∼50 µM) – were observed (Table 1), indicating that isoNAM inhibition does not occur through base exchange inhibition but rather through traditional competitive inhibition; i.e., isoNAM directly competes with NAD^+^ for binding to SIRT3. Prior literature [24] has indicated that isoNAM is not a base exchange substrate. This allows us to estimate *K_d_* for isoNAM using its *K_i_* obtained from fitting of equation (2).

Using the *K_i_* for isoNAM reported in Table 1 as an estimate of *K_d,isoNAM_* for SIRT3, the corresponding protein-inhibitor Gibbs free energy of binding may be estimated using 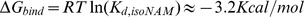. Note again that this is not intended to be an accurate estimate of *K_d,isoNAM_*, due to the approximations applied, but rather only its order of magnitude for the purpose of mechanistic analysis. A complete kinetic model for isoNAM inhibition is under development and more accurate estimation of *K_d,isoNAM_*, and *ΔG_bind,isoNAM_* will be considered in future work.

**Figure 12:**
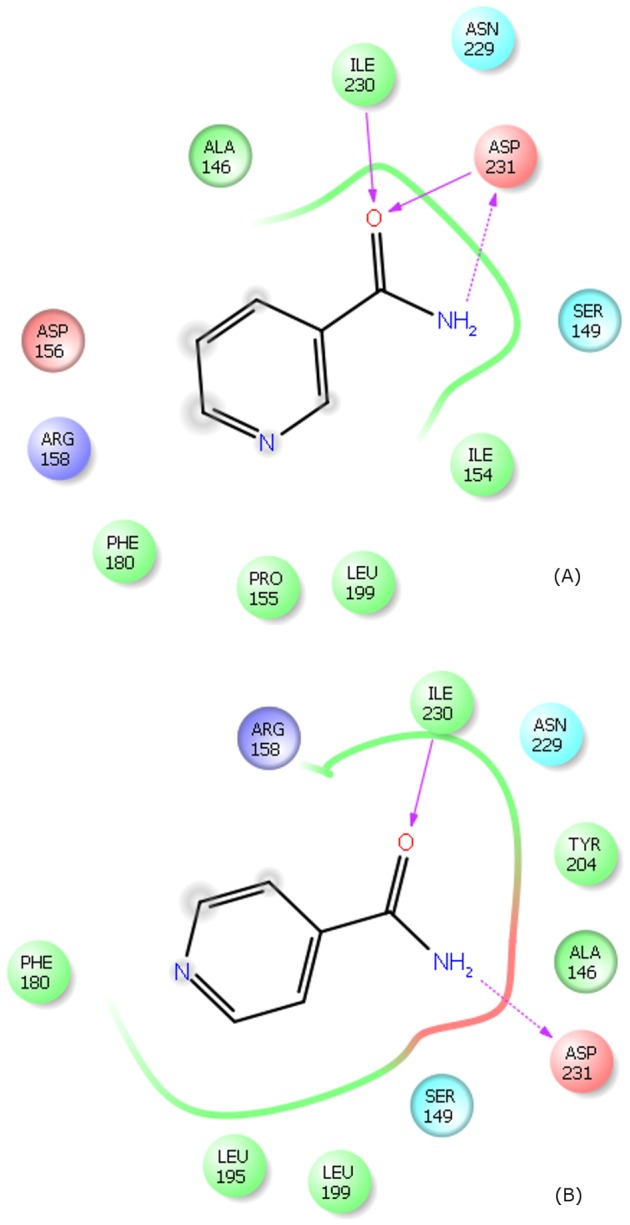
NAM/isoNAM interaction diagrams of MD averaged structures. (10 frames from last 10 ps) (**A**) SIRT3 complex with NAM; (**B**) SIRT3 complex with isoNAM.

Fig. 9(A) compares the binding modes of NAM and isoNAM in SIRT3 from MD simulations. Interaction diagrams in Fig. 12 depict the relevant contacts between C pocket residues and these ligands. Note that both NAM and isoNAM engage in favorable hydrogen bonding interactions through their amide groups with the side chain of Asp231 and backbone of Ile230. Similar hydrogen bonding interactions are observed in the complex of NAM with Sir2Tm (Fig. S1). The MM-PB(GB)SA binding affinity estimates are slightly more favorable for NAM compared to isoNAM (−19.82±1.89 kcal/mol *vs* −15.68±2.48 kcal/mol MM-GBSA, respectively), but the difference lies within the standard errors of the MM-GBSA estimates.

Since MM-GBSA estimates can generally be used to rank order binding affinities [60], and since the difference of MM-GBSA binding affinity estimates for NAM and isoNAM is close to the standard errors of the estimates, we adopt *K_d,isoNAM_* as an upper bound on *K_d,NAM_*. (We note that the difference between the C pocket binding affinities of NAM in the SIRT3:ADPR-Pr-Im:NAM and SIRT3:Ac-Pr:NAD^+^:NAM complexes lies within the standard errors of the estimates; hence we assume that this upper bound also applies to *K_d,NAM_* of the SIRT3:ADPR-Pr-Im:NAM complex.) Applying the *k_cat_<<k_j_*, *j≠cat* approximation discussed above for 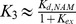, this also provides an approximate upper bound on the value of *K_ex_* for SIRT3:
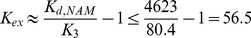
(7)which is consistent with the shape of the Dixon plots in Fig. 3. Initial rate data at higher [*NAM*] (not reported here), base exchange experiments [23] and QM/MM simulations can be used to improve the estimate of *K_ex_*. More accurate binding affinity estimates for NAM can also be obtained using linear interaction methods applied to a congeneric series [61] of C pocket binding ligands.

### Low NAD^+^ Binding Affinity is Consistent with Distinctive Kinetic Features of SIRT3 Inhibition by NAM

In the previous section, we showed that computational binding affinity estimates predict that the *K_d_* of NAM for SIRT3 is of the same order of magnitude as that of its isostere isoNAM. On the other hand, the *IC_50_* of NAM is at least two orders of magnitude lower than that of isoNAM. Together, these results imply that the potent product inhibition of SIRT3 by NAM observed at physiological concentrations should occur primarily through promotion of the base exchange reaction. In this section, we consider the other quantity in equations (3) and (6) that can be estimated through computational binding affinity calculations – namely, 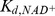 – and its implications for the mechanism of NAM inhibition of SIRT3.

Comparison of the computationally estimated binding affinities of NAD^+^ and NAM for SIRT3 in Tables 2 and 3, respectively, show that NAD^+^ binds to SIRT3 substantially more strongly than does NAM, rendering direct competition between NAM and NAD^+^ negligible when these molecules are present at similar concentrations. This can be attributed to the fact that the majority of favorable contacts with NAD^+^ occur with the ADPR moiety in the A pocket – including hydrogen bonding interactions between Ser321, Thr320 and the phosphate groups, as well as between Asn244, Arg345 and the ribose hydroxyl groups – instead of with the nicotinamide moiety in the C pocket.

Given the above evidence that base exchange is responsible for the observed NAM inhibition kinetics of SIRT3, we now consider the energetic origins of the differences between SIRT3 and SIRT1/Sir2 inhibition kinetics. Equation (6) indicates that under the approximation *k_cat_<<k_j_*, *j≠cat*, *α* depends on 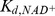, with more competitive character (greater 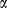) corresponding to a higher ratio 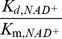 (note that 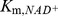 is also a function of 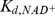). Moreover, according to equation (5), which is derived from our proposed kinetic model under the approximation *k_cat_<<k_j_*, *j≠cat*, the x-coordinate of the intersection point of the double reciprocal plots at various [*NAM*] provides an estimate of 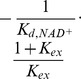 for a sirtuin. In this regard, the energetics of binding of NAD^+^ to the catalytically productive AC pocket in Sir2 and SIRT3 can be compared computationally. The MM-PB(GB)SA binding affinity estimates in Table 2 (−105.48±6.48 *vs* −65.63±5.91 kcal/mol by MM-GBSA, for Sir2Tm and SIRT3, respectively) indicate a significantly higher 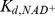 for SIRT3, which is consistent with the distinctive features of SIRT3's kinetics of inhibition by NAM, including the position of the intersection point in Fig. 2.

**Figure 13:**
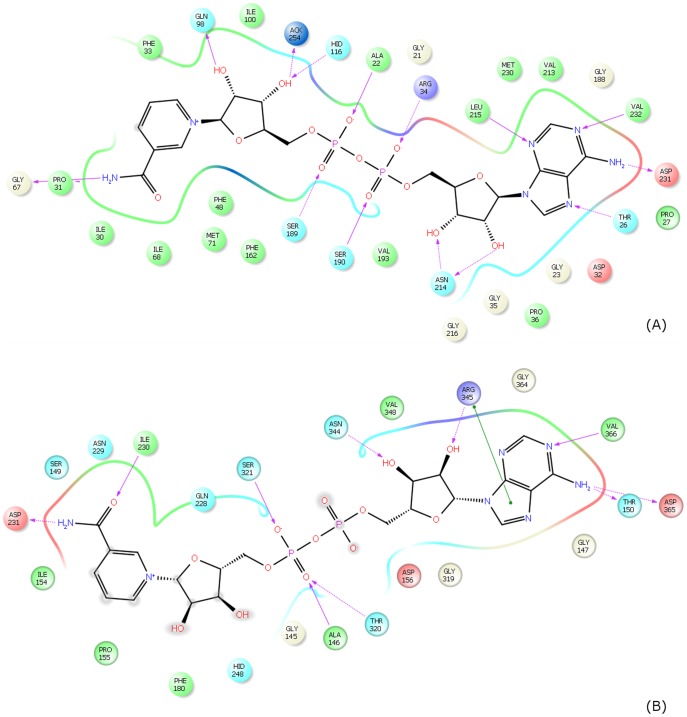
NAD^+^ interaction diagrams. (**A**) Sir2Tm-NAD^+^ interaction diagrams of MD averaged structures (10 frames from last 10 ps) - ternary complex (Sir2Tm:Ac-p53:NAD^+^); (**B**) SIRT3-NAD^+^ interaction diagrams of MD averaged structures (10 frames from last 10 ps) - ternary complex (SIRT3:Ac-CS2:NAD^+^).

The following features contribute to the significantly higher 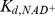 for SIRT3. Comparing the breakdown of binding energy contributions for SIRT3 and Sir2Tm in Table 2, the MD averaged structures in Fig. 7, 9(A) and the ligand interaction diagrams in Fig. 13, we see why NAD^+^ has stronger binding affinity for Sir2Tm in the ternary structure. The main contribution to the stronger binding affinity is the stronger electrostatic interaction between NAD^+^ and the sirtuin enzyme represented in the ΔEEL term of the energy component analysis shown in Table 2. The proximity of charged groups (e.g. Arg34 in Sir2Tm and Asp156 in SIRT3) near the negatively charged pyrophosphate moiety or positively charged nicotinamide moiety favors Sir2Tm:NAD^+^ binding, as observed from the interaction diagram between NAD^+^ and the enzyme-peptide complex constructed from the averaged structure from the last 10 ps of MD simulation. Other polar interactions such hydrogen bonds also favor Sir2Tm:NAD^+^ binding. In addition, in the Sir2Tm ternary structure, there are more hydrogen bond interactions formed between NAD^+^ and the receptor. It can also be found in the structural presentation of the binding sites. Also notable are the solvation energy losses. SIRT3 has less loss in solvation energy upon NAD^+^ binding in the ternary complex (Table 2). The greater solvent exposure of NAD^+^ in the SIRT3 complex can also be seen from the interaction diagrams of Fig. 13. However, the favorable solvation energy also leads to fewer favorable contacts between NAD^+^ and the enzyme/peptide substrate, resulting in lower binding affinity.

Thus, our computational estimates of 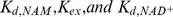 are all consistent with the proposed kinetic model (3) for sirtuin inhibition that relates these physiochemical quantities to the base exchange inhibition kinetics of sirtuins.

In some enzymatic reactions, given a fixed value of *k_cat_*, a larger *K_d_* of the substrate, implies a higher ratio *K_d_/K_m_*. This is not necessarily the case for all sirtuins. The values of other rate constants, including the exchange rate constants, can play an important role in determining 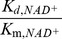 the value of *α*, and hence the extent of competition in NAM inhibition. Thus, further studies exploring the extent to which weak NAD^+^ binding correlates with competition in NAM inhibition kinetics are warranted and underway.

### Comparison of NAD^+^ Binding Affinities in Catalytically Productive and Unproductive Binding Modes: Implications for the Mechanism of NAM Inhibition of Deacetylation

As indicated above, there are two possible explanations for the greater degree of apparent competition between NAM and NAD^+^ in the inhibition kinetics of hSIRT3/mSIRT3, compared to that of SIRT1. These are a) base exchange (which increases the rate of the reverse reaction) with suitable values of 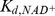 and 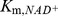 and b) direct competition (which reduces the rate of the forward reaction). In the previous sections, we have presented strong evidence for an explanation in terms of mechanism a). We now consider mechanism b).

Models for NAM inhibition of the forward deacetylation reaction that can accommodate the possibility of mixed noncompetitive inhibition kinetics have been proposed previously [42]. These involve NAD^+^ binding in the catalytically unproductive AB mode in the presence of NAM, with the cofactor's nicotinamide moiety undergoing a rapid conformational shift to the C pocket upon the dissociation of NAM. Noncompetitive inhibition could occur in this model if the binding affinities of NAD^+^ to the AB and AC pockets were roughly equal. Mixed noncompetitive inhibition with 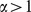 could occur within the model if the binding affinity of NAD^+^ for the AC pocket were higher than its binding affinity for the AB pocket.

A necessary condition for this mechanism to be a possible explanation for the differences in observed inhibition kinetics of SIRT1/Sir2 and SIRT3 is thus that NAD^+^ must bind with high affinity to the catalytically inactive (AB) pocket in SIRT1 and Sir2, but not SIRT3, concurrently with NAM binding. Hence this hypothesis can also be investigated with computational binding affinity estimation.

Figs. 6 and 7 depict the structural averages of the last 10 ps of the MD trajectories for the Sir2Tm:Ac-CS2:NAD^+^ (catalytically productive) and Sir2Tm:Ac-CS2:NAD^+:^NAM (catalytically unproductive) complexes, respectively. The corresponding MD structural averages for SIRT3 are shown in Fig. 9(A) and (B). Note that neither the Sir2Tm nor the SIRT3 catalytically unproductive binding modes shown in these Figures have been identified crystallographically in the presence of bound peptide [22], [40]. In the presence of NAM, the nicotinamide moiety of NAD^+^ in Sir2Tm and SIRT3 (Fig. 7 and 9A, respectively) does not engage in both the hydrogen bonding interactions observed for NAM in the MD averaged structures (Fig. 12). The B pocket is more solvent-exposed, and contains fewer potential hydrogen bonding partners than the C pocket in SIRT3 and Sir2Tm. Comparison of the MM-GB(PB)SA binding affinity estimates for these complexes in Tables 3 and 4 reveals that in Sir2Tm, the catalytically unproductive binding mode is predicted to be less stable than the productive binding mode (−81.78±8.01 kcal/mol *vs* −105.48±6.48 kcal/mol MM-GBSA, respectively). Moreover, in SIRT3, the estimated binding affinity of NAD^+^ for the AB pocket (−63.12 kcal/mol MM-GBSA) is similar to that for the AC pocket (−65.63 kcal/mol). Hence, direct competition between NAM and NAD^+^ is not a viable explanation for the observed competitive behavior in the NAM inhibition kinetics for SIRT3, and the difference in 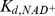
's for the catalytically productive binding modes of these two enzymes, discussed above, is much more relevant for explaining the distinctive features of SIRT3 inhibition kinetics.

These observations further substantiate our proposed model (Eq. 6) explaining the observed competitive behavior in NAM inhibition of SIRT3 in terms of base exchange and the energetics of NAD^+^ binding to the catalytically productive AC pocket.

### Prospects for Activation of SIRT3 by Derepression of NAM Inhibition

Activation of sirtuins has been the subject of intense pharmacological interest [23], [62]–[65] due to its implications for the treatment of aging and age-related diseases. However, understanding of the scope for activation of mammalian sirtuins is limited. Structure-based design of allosteric sirtuin activators [63] is challenging. Structure-based design of activators that operate via NAM derepression is possible, but requires a detailed model of the inhibition mechanism. In this section, we consider the implications of the base exchange inhibition mechanism of SIRT3 elucidated herein for pharmacological activation of this enzyme through NAM derepression.

Like Sir2, SIRT3 is potently inhibited by NAM. However, the mechanistic details of base exchange play a critical role in determining the scope of possible strategies that can activate SIRT3 by derepression of NAM inhibition. In these strategies, competitive inhibition of the base exchange reaction must be possible without substantial competitive inhibition of the deacetylation reaction.

Sauve et al. [24] provided an estimate for the base exchange equilibrium constant in mSIRT1 of about 20. Based on this estimate alone, the maximal possible activation of mSIRT1 by complete inhibition of the base exchange reaction at a given NAM concentration is higher than that of yeast Sir2, which has been successfully activated by competitive inhibitors of the base exchange reaction like isoNAM [23]. As noted above, isoNAM is a ligand that can compete with NAM for binding but cannot initiate the reverse reaction, thereby leading to apparent activation through relief of nicotinamide inhibition [23], [66]. In the present work, we have provided an upper bound (Eq. 7) on *K_ex_* of SIRT3 that is consistent with the possibility of activation of this enzyme by derepression of NAM inhibition. We have also provided the first report of the effect of NAM derepression on the activity of a mammalian sirtuin.

The data in Fig. 5 show that under the current experimental conditions, addition of 900 µM isoNAM only slightly decreases the extent of SIRT3 inhibition in the presence of 100 µM NAM. In this regard, the information provided by NAM inhibition kinetics (e.g., the value of *α*) regarding the relative magnitudes of binding rate constants and other (e.g., base exchange) rate constants in the reaction mechanism has implications for SIRT3 activation by NAM derepression. An inert C pocket binding ligand like isoNAM will further increase the apparent 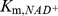 of the enzyme, and hence the value of 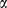 for NAM inhibition, which quantifies the effective competition between NAM and NAD^+^ in the absence of activator, will affect the scope and strategy for sirtuin activation through base exchange derepression. We have shown that base exchange product inhibition of SIRT3 displays a high value of *α*, which may originate in differences between 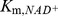 and 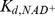. The difference between the 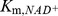 and 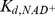 of a sirtuin may therefore be an important characteristic of its catalytic and inhibitory mechanisms that has implications for pharmacological activation, and should be explored further. Steady state models are essential tools for understanding these differences and will be developed further in future work. Future work should also involve QM/MM simulations and base exchange experiments to more accurately estimate *k_ex_*, *k_−ex_* of mammalian sirtuins. More accurate binding affinity estimates for C pocket binding ligands can also be made using linear interaction methods on congeneric series [61].

Prior work on NAM derepression correctly indicated that the maximal possible activation of mammalian sirtuins is in some cases greater than that of yeast and bacterial sirtuins. Indeed, SIRT1 and SIRT3 have evolved high *K_ex_*'s, which result in the possibility of nearly complete inhibition by NAM at concentrations significantly below *K_d,NAM_* – hence allowing them to serve as good NAM sensors at low physiological concentrations of NAM. However, these mammalian sirtuins have also evolved higher 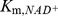
's in order to function as NAD^+^ sensors. Prior work on NAM derepression did not consider the implications of the higher 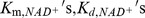 and different degrees of competitive behavior in the NAM inhibition kinetics of mammalian sirtuins and yeast/bacterial sirtuins for the scope and design criteria for sirtuin activation by NAM derepression. The mechanistic understanding provided herein may therefore be useful for clarifying the feasibility of a particular activation strategy.

Recent evidence suggests that certain symptoms of aging can be reversed in mammals by sirtuin upregulation. In several reports (e.g., [16]), sirtuin upregulation was achieved by increasing NAD^+^ levels. Reducing NAM inhibition may also be a viable strategy for activating certain mammalian sirtuins. Development of initial rate models for the sirtuin catalytic cycle based on methods similar to those presented in this work will serve as an essential foundation for the rational computational design of sirtuin activators that operate through NAM derepression. By coupling to an appropriate computational model for binding affinity prediction based on MM-PB(GB)SA, computationally driven activator design may be possible. Similar methods could be applied to computationally screen for C pocket mutations that modulate base exchange inhibition through modification of *K_d,NAM_*, for the purpose of understanding the selection pressures that drove the evolution of the diverse sensitivities of sirtuin enzyme active sites to NAM [67], [68].

## Conclusions

In this paper, we have presented experimental data demonstrating differences in the NAM inhibition kinetics of SIRT3 and SIRT1 that have important implications for both the physiological regulation of these enzymes in the “NAD^+^ world” and the prospects for their pharmacological activation by relief of base exchange inhibition. We developed and reported a kinetic model for sirtuin base exchange inhibition by NAM that is capable of relating differences in inhibition kinetics of sirtuins to the physicochemical properties of the enzymes, in particular their 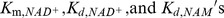. We have also presented the first computational estimates for the 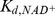 and 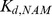 of SIRT3 and demonstrated how aspects of the experimentally observed differences in inhibition kinetics of SIRT1 and SIRT3 can be explained in terms of the binding affinity of NAD^+^. This model and approach can be applied to any sirtuin and hence provides a framework for a) understanding the physicochemical origins of the diversity of mixed noncompetitive inhibition kinetics of the mammalian sirtuins SIRT1-7 and b) prediction of the effects on these enzymes of sirtuin activators that operate through derepression of base exchange inhibition.

## Materials and Methods

### Chemicals and Reagents

The acetylated substrate peptide based on the sequence of Acetyl-coenzyme A synthetase 2 (AceCS2 638–649, H2N-TRSGK (Ac)VMRRLLR-OH) was synthesized at PEPTIDE 2.0 Inc(Chantilly, VA, USA). Human recombinant SIRT3 was purchased from Creative BioMart(Shirley, NY, USA). Enzyme concentrations were determined using the method of Bradford [69] with bovine serum albumin (BSA) as the standard. All other chemicals used were of the highest purity commercially available and were purchased from Sigma (St. Louis, MO, USA), and Fisher Scientific (Pittsburgh, PA, USA).

### Measurement of Deacetylation Activity Using a Fluorolabeled Peptide

The steady state parameters (*K_m_* and *k_cat_*) and catalytic efficiency (*k_cat_/K_m_*) of deacetylase activity of recombinant human SIRT1 and SIRT3 were determined using a fluorometric assay. The deacetylation activities were measured by using the SIRT3 Fluorimetic Drug Discovery Kit (AK 557, Enzo Life Sciences) and SIRT1 Fluorimetric Drug Discovery Kit (AK 555, Enzo Life Sciences). This assay system allows detection of a fluorescent signal upon deacetylation of an acetylated substrate peptide, comprising amino acids 317–320 of human p53 (Gln-Pro-Lys-Lys^Ac^) for SIRT3 and 379–382 (Arg-His-Lys-Lys^Ac^) for SIRT1, when treated with developer. The intensity of fluorescence was measured on a fluorometric microplate reader (Fluoroskan Ascent® FL, Thermo LabSystems) with excitation set at 355 nm and emission detection set at 460 nm. The initial rate of the NAD^+^-dependent deacetylation activity of SIRT3 enzyme was measured at different concentrations of NAD^+^. The reactions were carried out at 37°C in a 50 µl reaction volume containing 50 mM Tris/Cl (pH = 8), 137 mM NaCl, and 100 µM fluorolabeled peptide substrate. Unless otherwise indicated, all initial rate measurements were means of three or more replicates, obtained with single incubation times, at which point 5% or less of the substrate initially present had been deacetylated. The raw data were fitted to the Michaelis-Menten equation and defined inhibition models (Eqs. 1 and 2) by using GraphPad Prism (GraphPad Software, Inc, CA) to obtain the kinetic constants. Fluorimetric assays of sirtuin activity have been shown to provide results comparable to those from assays using unmodified peptides in studies of nonallosteric modulators [54], [70]. In assays of allosteric modulators – which are not considered in the present work – artifacts reported in the presence of the fluorescent label [71] were later shown to occur due to the hydrophobic fluorophore participating in the modulator's allosteric activation mechanism [72]. In the present work, mechanistic conclusions drawn from deacetylation kinetics data were further corroborated through computational modeling of the active site (see below).

### Measurement of *IC*_50_Values for SIRT3 and SIRT1 Inhibitors NAM and isoNAM

This assay was used to measure the potency of inhibition of SIRT3 and SIRT1 by NAM, and isoNAM. Reactions were performed in the presence of 100 µM NAD^+^, and either NAM (0, 1, 5, 10, 50, 100, 200, 500 µM) or isoNAM (0, 0.05, 0.1, 1, 5, and 10 mM). The initial rates were measured at different concentrations of NAM and isoNAM, and the reaction conditions were the same as above. The data were fitted to equation (8) by using Prism to calculate the *IC*_50_ values:
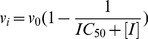
(8)where *v_0_* is the initial rate of the uninhibited reaction and *v_i_* is the initial rate of the reaction at concentration I of the inhibitor.

### Measurement of the Relative Inhibition Effect of IsoNAM on SIRT3 in the presence of NAM

The deacetylation activity was measured by using the SIRT3 Fluorimetric Drug Discovery Kit (AK 557, Enzo Life Sciences). In the presence of 100 µM of NAM, deacetylation activity of SIRT3 enzyme was measured under addition of different concentrations of isoNAM, range from 50 µM to 900 µM. The reactions were carried out at 37°C in a 50 µl reaction volume containing 50 mM Tris/Cl (pH = 8), 137 mM NaCl, and 100 µM fluorolabeled peptide substrate.

### Molecular Dynamics Simulations of Ligand Binding

The crystal structure of SIRT3/Ac-CS2/Carba-NAD^+^ (PBDID: 4FVT) was used as the starting structure to construct all the structures in SIRT3 MD simulations. The ternary complex was constructed by simply modifying Carba-NAD^+^ to NAD^+^ and replacing norleucine with methionine as in the original sequence of Ac-CS2. The SIRT3/NAD^+^ binary structure was obtained by removing the peptide substrate from the ternary complex. For the complex also including nicotinamide as inhibitor, NAD^+^ and NAM coordinates were taken directly from the crystal structure of the Sir2Af2/NAD^+^ complex (PBDID: 1YC2 chain A) after structural alignment between 4FVT and 1YC2:A. The complex including iso-nicotinamide was built by modifying NAM to isoNAM in the above structure. All other molecules including waters were removed. Extra sodium and chloride ions were added to neutralize the system and maintain an ionic strength of about 0.06 M, followed by solvation using boxes of TIP3P water molecules with a margin of 12.0 Å from solute atoms in all three dimensions.

Models for SIRT3, Ac-CS2 and NAD^+^ alone were also constructed by taking the coordinates from 4FVT crystal structure, followed by the same neutralization and solvation process.

The crystal structure of Sir2Tm:Ac-p53:NAD^+^ (PBDID: 2H4F) was used as the starting structure to construct all the structures in SIRT3 MD simulations. The missing loop of residue 37–42 was modeled using an *ab initio* loop prediction method in Prime version 3.2 (Schrödinger, LLC) during the protein preparation stage. The Sir2Tm:NAD^+^ binary structure was obtained by removing the peptide substrate from the ternary complex. The complex of Sir2Tm:Ac-p53:NAD^+^:NAM was obtained by first removing the NAD^+^, followed by superimposing the NAD^+^ and NAM from 1YC2:A after structural alignment. All other molecules including waters were removed, and extra sodium and chloride ions were added to maintain system neutrality. The complexes were then solvated using boxes of TIP3P water molecules with a margin of 12.0 Å from solute atoms.

The Amber99SB force field [73], [74] was used for all the molecular mechanics calculations. Extra parameters were adapted from the following sources: parameters for Zn developed by Luo's group [75]; parameters for NAD^+^ developed by Walker et al. [76] and Pavelites et al. [77]; parameters for acetylated lysine developed by Papamokos et al. [78]. Charges for NAM and isoNAM were derived from RESP fits to electrostatic potentials obtained at HF/6-31(d) level of theory that is consistent with the Amber99SB force field.

All MD simulations were performed with periodic boundary conditions to produce isothermal-isobaric ensembles (NPT) at 300 K using the NAMD program [79]. The Particle Mesh Ewald (PME) method [80] was used to calculate the electrostatic energy. The covalent bonds involving hydrogen atoms were frozen with the SHAKE algorithm [81]. Temperature was regulated using the Langevin dynamics with the collision frequency of 1 ps^−1^. Pressure regulation was achieved with isotropic position scaling and the pressure relaxation time was set to 1.0 picosecond. The integration of the equations of motion was conducted at a time step of 2 femtoseconds.

There were three phases in the MD simulations. First, in the relaxation phase, the system underwent a 2000-step minimization before a short 200 ps NPT MD simulation, with the main chain atoms of the protein restrained to the positions in the crystal structures with force constants of 5 kcal mol^−1^ Å^−2^. Next, the systems ran for various lengths of time up to 22 ns in the equilibration phase. Last, the sampling phase included a 10 ns of MD simulation.

### Binding Affinity Estimation

Binding free energies were calculated using the MM-PBSA and the MM-GBSA methods as implemented in the AMBER package [82]. In MM-PBSA and MM-GBSA, binding free energy is evaluated as:
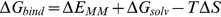
(9)where *ΔE*_MM_, *ΔG*_solv_ and *TΔS* are the changes of gas-phase interaction energy, solvation free energy, and solute conformational entropy change upon binding, respectively. *ΔE*_MM_ includes internal energy in bonded terms, electrostatic and van der Waals energies. *ΔG*_solv_ is the sum of polar contributions calculated using the PB or GB model, and nonpolar contributions estimated from solvent-accessible surface area (SASA).

All the calculations were carried out using the MMPBSA.py module with AmberTools13 [82]. The polar contribution of the solvation free energy was calculated by the GB model developed by Onufriev et al. [83] and by the PB method implemented in the pbsa program. A salt concentration of 0.1 M was used in MM-GBSA calculations. The solvent-accessible surface area was evaluated using the LCPO method [84]. Because relative free energy trends were of interest, solute conformational entropy change was neglected. Energies were evaluated using 10000 snapshots extracted from the last 10 ns at a time interval of 1 ps for each trajectory after ensuring that each one of these trajectories was completely stable. One exception is the highly dynamic SIRT3/NAD^+^ binary system, where 20000 snapshots from last 20 ns were used in energy evaluations.

## Supporting Information

Figure S1 **NAM interaction diagrams of MD averaged structures (10 frames from last 10 ps) of Sir2TM complex with NAM.**
(PDF)
