# Correction: Mechanism of Inhibition of the Human Sirtuin Enzyme SIRT3 by Nicotinamide: Computational and Experimental Studies

**DOI:** 10.1371/journal.pone.0136127

**Published:** 2015-08-14

**Authors:** Xiangying Guan, Ping Lin, Eric Knoll, Raj Chakrabarti

The authors would like to provide the expressions for K_1_,K_2_,K_3_, K_m,NAD+_ and v_max_ for Equation 3, which should have been included as a supplementary file with the article.

The expressions for K_1_,K_2_,K_3_,K_m,NAD+_ and v_max_ are provided in S1 File in this Correction.

For sirtuins, *k_cat_<< k_j_, j ≠ cat* does not imply *K_m, NAD+_ ≈ K_d, NAD+_*; see S1 File.

## Supporting Information

S1 File Initial rate model for sirtuin deacetylation kinetics. (PDF)

## References

[1] Guan X, Lin P, Knoll E, Chakrabarti R (2014) Mechanism of Inhibition of the Human Sirtuin Enzyme SIRT3 by Nicotinamide: Computational and Experimental Studies. PLoS ONE 9(9): e107729 doi: 10.1371/journal.pone.0107729 25221980 10.1371/journal.pone.0107729 PMC4164625

