# Correction: Correction: Mechanism of Inhibition of the Human Sirtuin Enzyme SIRT3 by Nicotinamide: Computational and Experimental Studies

**DOI:** 10.1371/journal.pone.0138393

**Published:** 2015-09-14

**Authors:** The PLOS ONE Staff

## Notice of Republication

This correction was republished on August 31, 2015, to replace an incorrectly uploaded file and include the correct supplementary file. The publisher apologizes for the error. Please download this correction again to view the correct version. The originally published correction and the republished correction are provided here for reference.

## Supporting Information

S1 PDF Originally published correction (PDF)

S2 PDF Republished correction (PDF)
