# Supplementary material for: Mechanism of Inhibition of the Human Sirtuin Enzyme SIRT3 by Nicotinamide: Computational and Experimental Studies
Source: PLoS One. 2014 Sep 15;9(9):e107729. doi: 10.1371/journal.pone.0107729 (PMC4164625; doi:10.1371/journal.pone.0107729)

Supporting Information

Figure S1. NAM interaction diagrams of MD averaged structures (10 frames from last 10ps) of Sir2Tm complex with NAM.

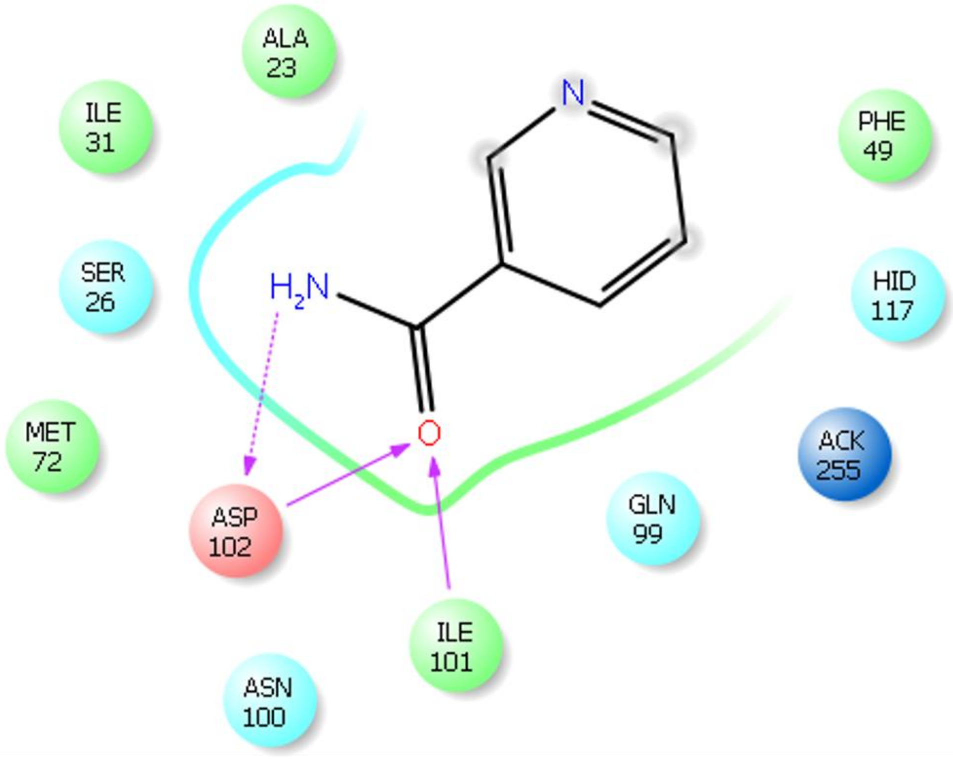

Supplement: Figure S1 — NAM interaction diagrams of MD averaged structures (10 frames from last 10 ps) of Sir2TM complex with NAM. (PDF) [file pone.0107729.s001.pdf]
