# Supplementary material for: Correction: Mechanism of Inhibition of the Human Sirtuin Enzyme SIRT3 by Nicotinamide: Computational and Experimental Studies
Source: PLoS One. 2015 Aug 14;10(8):e0136127. doi: 10.1371/journal.pone.0136127 (PMC4537223; doi:10.1371/journal.pone.0136127)
Supplement: S1 File — (PDF) [file pone.0136127.s001.pdf]

Addendum S1. Initial rate model for sirtuin deacetylation kinetics

The following initial rate law, derived using steady state assumptions, was used for fitting deacetylation kinetics of sirtuins in the presence of NAM:

$$\frac{v}{v_{\max}} = \frac{[\mathrm{NAD^+}]\left(1+\frac{[\mathrm{NAM}]}{K_1}\right)}{K_{m,\mathrm{NAD^+}}\left(1+\frac{[\mathrm{NAM}]}{K_2}\right)+[\mathrm{NAD^+}]\left(1+\frac{[\mathrm{NAM}]}{K_3}\right)}$$

with

$$\begin{aligned} v_{\max} &= \frac{k_{cat} * \mathrm{k_1 k_{ex} k_{-2}}\left(k_{-2}+k_{cat}\right)}{k_{-2}k_1k_{ex}k_{-2}+k_{cat}\left(k_{-2}k_{cat}k_1+k_{-2}k_{-2}k_1+k_{-2}k_1k_{-ex}+k_{-2}k_1k_{ex}+k_1k_{ex}k_{cat}\right)}[E]_0 \\ &\approx k_{cat}[E]_0 \end{aligned}$$

$$\begin{aligned} K_{m,NAD^+} &= \frac{k_{cat}k_{-2}\left[k_{ex}k_{cat}+k_{-1}k_{cat}+k_{ex}k_{-2}+k_{-1}k_{-2}+k_{-ex}k_{-1}\right]}{k_{-2}k_1k_{ex}k_{-2}+k_{cat}\left(k_{-2}k_{cat}k_1+k_{-2}k_{-2}k_1+k_{-2}k_1k_{-ex}+k_{-2}k_1k_{ex}+k_1k_{ex}k_{cat}\right)} \\ &\approx k_{cat}\frac{k_{ex}k_{-2}+k_{-1}\left(k_{-2}+k_{-ex}\right)}{k_1k_{ex}k_{-2}} = k_{cat}\left(\frac{1}{k_1}+K_{d,NAD+}\frac{k_{-2}+k_{-ex}}{k_{-2}k_{ex}}\right) \end{aligned}$$

$$\frac{1}{K_1}=\frac{k_2}{k_{-2}+k_{cat}}\approx\frac{1}{K_{d,NAM}}$$

$$\frac{1}{K_2}=\frac{1}{K_{m,\mathrm{NAD^+}}}\frac{k_2k_{-ex}k_{-1}k_{-2}+k_{cat}\left(k_2k_{ex}k_{cat}+k_{-1}k_2k_{cat}+k_{-ex}k_{-1}k_2+2k_{-2}k_2k_{ex}+2k_{-2}k_2k_{-1}\right)}{k_{-2}k_1k_{ex}k_{-2}+k_{cat}\left(k_{-2}k_{cat}k_1+k_{-2}k_{-2}k_1+k_{-2}k_1k_{-ex}+k_{-2}k_1k_{ex}+k_1k_{ex}k_{cat}\right)}\approx\frac{K_{d,NAD^+}K_{ex}}{K_{m,\mathrm{NAD^+}}K_{d,NAM}}$$

$$\frac{1}{K_3}=\frac{1}{\alpha K_2}=\frac{k_1k_2k_{-2}\left(k_{-ex}+k_{ex}\right)+k_{cat}k_1k_2\left(k_{-2}+k_{ex}\right)}{k_1k_{-2}k_{-2}k_{ex}+k_{cat}\left(k_{-2}k_{cat}k_1+k_{-2}k_{-2}k_1+k_{-2}k_1k_{-ex}+k_{-2}k_1k_{ex}+k_1k_{ex}k_{cat}\right)}\approx\frac{1+K_{ex}}{K_{d,NAM}}$$

where the approximations (applied in equation (3) in the text) refer to the case where

$k_{cat} \ll k_j,\ j \neq cat$
