# Supplementary material for: Correction: Correction: Mechanism of Inhibition of the Human Sirtuin Enzyme SIRT3 by Nicotinamide: Computational and Experimental Studies
Source: PLoS One. 2015 Sep 14;10(9):e0138393. doi: 10.1371/journal.pone.0138393 (PMC4569365; doi:10.1371/journal.pone.0138393)
Supplement: S2 PDF — (PDF) [file pone.0138393.s002.pdf]

PLOS ONE

CORRECTION

Correction: Mechanism of Inhibition of the Human Sirtuin Enzyme SIRT3 by Nicotinamide: Computational and Experimental Studies

Xiangying Guan, Ping Lin, Eric Knoll, Raj Chakrabarti

The authors would like to provide the expressions for K_1,K_2,K_3, $K_{m,NAD+}$ and v_{max} for Equation 3, which should have been included as a supplementary file with the article.

The expressions for K_1,K_2,K_3,$K_{m,NAD+}$ and v_{max} are provided in S1 File in this Correction.

For sirtuins, $k_{cat} << k_j, j \neq cat$ does not imply $K_{m,\ NAD+} \approx K_{d,\ NAD+}$; see S1 File.

Supporting Information

S1 File. Initial rate model for sirtuin deacetylation kinetics.
(PDF)

Reference

1. Guan X, Lin P, Knoll E, Chakrabarti R (2014) Mechanism of Inhibition of the Human Sirtuin Enzyme SIRT3 by Nicotinamide: Computational and Experimental Studies. PLoS ONE 9(9): e107729. doi: 10.1371/journal.pone.0107729 PMID: 25221980

CrossMark
click for updates

OPEN ACCESS

Citation: Guan X, Lin P, Knoll E, Chakrabarti R (2015) Correction: Mechanism of Inhibition of the Human Sirtuin Enzyme SIRT3 by Nicotinamide: Computational and Experimental Studies. PLoS ONE 10(8): e0136127. doi:10.1371/journal.pone.0136127

Published: August 14, 2015

Copyright: © 2015 Guan et al. This is an open access article distributed under the terms of the Creative Commons Attribution License, which permits unrestricted use, distribution, and reproduction in any medium, provided the original author and source are credited.
